# Revealing Ion Adsorption and Charging Mechanisms in Layered Metal–Organic Framework Supercapacitors with Solid-State Nuclear Magnetic Resonance

**DOI:** 10.1021/jacs.4c05330

**Published:** 2024-08-12

**Authors:** Chloe J. Balhatchet, Jamie W. Gittins, Seung-Jae Shin, Kangkang Ge, Xinyu Liu, Teedhat Trisukhon, Shivani Sharma, Thomas Kress, Pierre-Louis Taberna, Patrice Simon, Aron Walsh, Alexander C. Forse

**Affiliations:** † Yusuf Hamied Department of Chemistry, University of Cambridge, Lensfield Road, Cambridge CB2 1EW, United Kingdom; ‡ Thomas Young Centre and Department of Materials, Imperial College London, London SW7 2AZ, United Kingdom; § CIRIMAT, UMR CNRS 5085, Université Paul Sabatier Toulouse III, Toulouse 31062, France; ∥ Department of Chemical and Biomolecular Engineering and Department of Chemistry, University of California, Berkeley, California 94720, United States; ⊥ RS2E, Réseau Français sur le Stockage Electrochimique de l’Energie, FR CNRS 3459, Amiens Cedex 80039, France

## Abstract

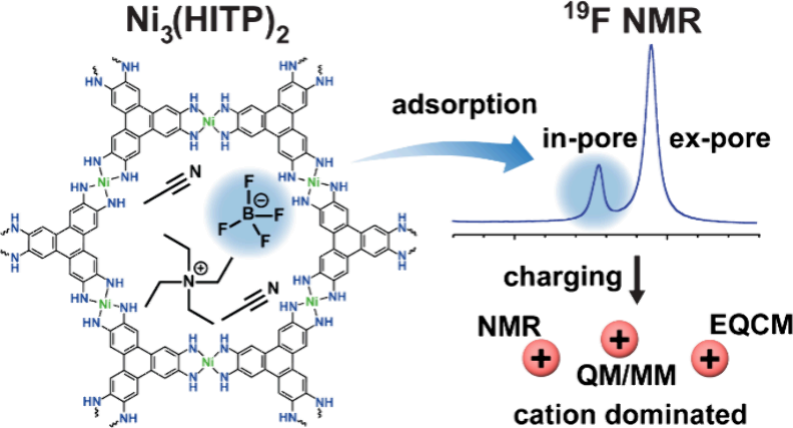

Conductive layered metal–organic frameworks (MOFs) have demonstrated promising electrochemical performances as supercapacitor electrode materials. The well-defined chemical structures of these crystalline porous electrodes facilitate structure–performance studies; however, there is a fundamental lack in the molecular-level understanding of charge storage mechanisms in conductive layered MOFs. To address this, we employ solid-state nuclear magnetic resonance (NMR) spectroscopy to study ion adsorption in nickel 2,3,6,7,10,11-hexaiminotriphenylene, Ni_3_(HITP)_2_. In this system, we find that separate resonances can be observed for the MOF’s in-pore and ex-pore ions. The chemical shift of in-pore electrolyte is found to be dominated by specific chemical interactions with the MOF functional groups, with this result supported by quantum mechanics/molecular mechanics (QM/MM) and density functional theory (DFT) calculations. Quantification of the electrolyte environments by NMR was also found to provide a proxy for electrochemical performance, which could facilitate the rapid screening of synthesized MOF samples. Finally, the charge storage mechanism was explored using a combination of *ex-situ* NMR and *operando* electrochemical quartz crystal microbalance (EQCM) experiments. These measurements revealed that cations are the dominant contributors to charge storage in Ni_3_(HITP)_2_, with anions contributing only a minor contribution to the charge storage. Overall, this work establishes the methods for studying MOF–electrolyte interactions via NMR spectroscopy. Understanding how these interactions influence the charging storage mechanism will aid the design of MOF–electrolyte combinations to optimize the performance of supercapacitors, as well as other electrochemical devices including electrocatalysts and sensors.

## Introduction

Conductive layered metal–organic frameworks (MOFs) are a class of crystalline materials that feature high intrinsic porosities and conductivities.^1^ These properties have led to promising applications across a diverse range of research fields including energy storage, electrochemical sensing, electrocatalysis, thermoelectrics, and spintronics.^2−9^ In particular, conductive MOFs of high porosity make ideal electrodes for energy storage in supercapacitors.^3,8,10^ The tunable structure of these materials, achieved through varying the identity of the constituent metal ions and organic linkers, presents an exciting opportunity for understanding molecular-level electrolyte–electrode interactions, and how these interactions impact electrochemical performance. This understanding will be essential for systematic optimization of electrochemical systems and to overcome the various challenges facing MOF-based supercapacitors, such as their limited charging rates compared to conventional activated carbon-based supercapacitors.^10^

Initial theoretical and experimental studies have begun to investigate the electrochemical interface of conductive layered MOFs and study charge storage in conductive MOFs. Bi et al. used molecular dynamics (MD) simulations to study the charge storage mechanism in MOFs with various pore sizes, including Ni_3_(HITP)_2_, with an ionic liquid electrolyte.^11^ This study offered the first insights into the ion distribution at the electrochemical interface for MOFs, revealing distinct distributions of in-pore cations and anions. Further computational studies have built on these foundations by using a hybrid quantum-mechanics/molecular-mechanics (QM/MM) approach to study the interface of the related layered MOF, copper 2,3,6,7,10,11-hexahydroxytriphenylene, Cu_3_(HHTP)_2_, with the benchmark organic electrolyte tetraethylammonium tetrafluoroborate in acetonitrile (NEt_4_BF_4_/ACN). This latter approach more accurately revealed the charge density distribution at the electric double-layer interface and was used to assess the favorability of different charging mechanisms for the system.^12^ The study suggested that predicted capacitance values for cation-dominated charging mechanisms matched most closely with experimental measurements for this system and that the preferred anion sorption sites were dependent on the electrode polarity.

Electrochemical quartz crystal microbalance (EQCM) experiments were used to experimentally study the same MOF–electrolyte system employed in the QM/MM study above, Cu_3_(HHTP)_2_ with 1 M NEt_4_BF_4_/ACN, and supported the cation-dominated nature of the charging mechanism.^13^ More widely, He et al. performed *in-situ* small-angle neutron scattering (SANS) experiments, on Ni_3_(HITP)_2_ cells with an organic electrolyte of sodium triflate in dimethylformamide (DMF).^14^ The charging mechanism was found to be dependent on the electrode polarization, and the MOF was proposed to be ionophobic with respect to this electrolyte, with the pores devoid of electrolyte ions in the absence of an applied potential, a scenario which has been predicted to lead to improved performance in nanoporous carbon supercapacitors.^15−17^ However, both SANS and EQCM rely on data fitting to separate electrolyte cation, anion, and solvent contributions, meaning it is challenging to quantify the charging mechanisms with these techniques. There is an urgent need for new model-free techniques that can directly study the independent interactions of cations and anions at the MOF interface and how this connects with the charging mechanism and performance.

Here, we propose the use of solid-state nuclear magnetic resonance (NMR) spectroscopy to reveal ion electrosorption in a layered MOF. NMR has been demonstrated to be a powerful technique to probe and quantify the electrolyte environments in porous carbon electrodes.^18,19^ In these materials, “in-pore” and “ex-pore” electrolyte environments can be identified from NMR spectra, where the chemical shift dependence of the in-pore resonances are dominated by ring-current effects.^20^ This leads to a characteristic nucleus-independent chemical shift (NICS) of the in-pore environment, which is shielded significantly relative to the neat electrolyte.^21^ Utilizing this assignment, charging mechanisms of porous carbon supercapacitors have been studied by measuring how the in-pore ion populations change upon charging through both *ex-situ* and *in-situ* NMR experiments.^18,22−25^ In contrast to EQCM and SANS, NMR can selectively and quantitatively probe cations, anions, and solvent species in the system by observing different nuclei and therefore has the potential to resolve some of the existing ambiguity in the literature on MOF electrodes. Indeed, NMR has already been used to identify and quantify “in-pore” molecular environments in nonconductive MOFs, where the in-pore chemical shift has been found to arise from a competing combination of coordination, solvation, and ring-current effects.^26,27^ NMR relaxometry has similarly demonstrated the potential to identify guest species in paramagnetic MOFs, as well as probing material porosities, providing several potential useful applications for NMR to be explored on porous conductive MOFs for electrochemical applications.^28−30^ Despite this, the application of NMR to study adsorption behavior of conductive layered MOFs remains unreported.

This study employs NMR for the first time to study the nature of the interaction between organic electrolytes and a conductive layered MOF, and demonstrates how these interactions might impact the charging mechanism. Solid-state NMR reveals separate in-pore and ex-pore electrolyte environments in Ni_3_(HITP)_2_, enabling the study of the electrochemical double layer separately from bulk electrolyte. Our experimental spectra alongside the QM/MM and density functional theory (DFT) calculations reveal that specific interactions between the MOF and the electrolyte dominate the observed chemical shifts, and we further find a correlation between measured in-pore ion populations and supercapacitor performance in different MOF batches. Finally, *ex-situ* NMR and *operando* EQCM experiments reveal the cation-dominated charging mechanism of the conductive MOF supercapacitor device. We suggest that exploring the interplay among MOF–electrolyte interactions, charging mechanisms, and performance could lead to the design of improved supercapacitor systems.

## Results and Discussion

### Synthesis and Characterization

**Figure 1:**
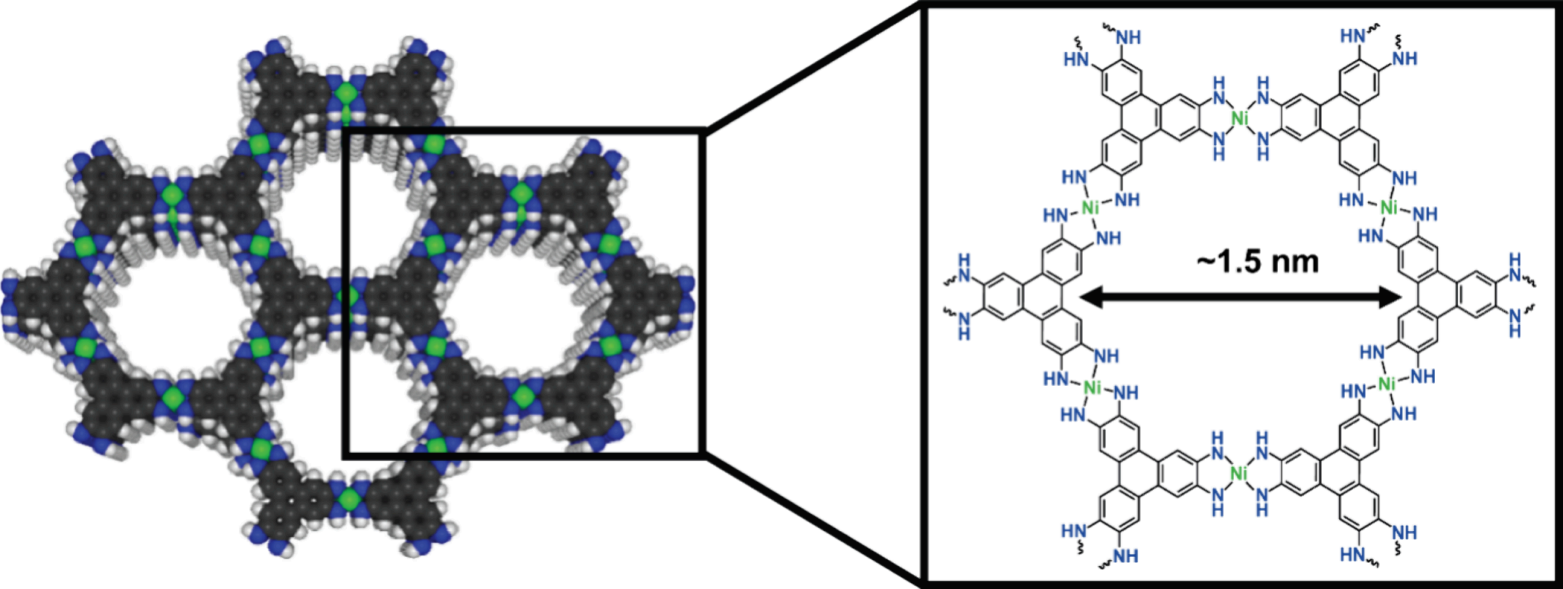
Structure of Ni_3_(HITP)_2_ with an enlarged Lewis structure of the hexagonal pore structure.

To study conductive layered MOF supercapacitors with NMR spectroscopy, nickel 2,3,6,7,10,11-hexaiminotriphenylene, Ni_3_(HITP)_2_, was selected as it had already been reported to have good supercapacitor performance and had been used in both experimental and computational studies of supercapacitor charging mechanisms (Figure 1).^5,11,14^ In this MOF, the Ni^2+^ sites are spd2 hybridized in a square planar configuration, with the resulting low-spin, diamagnetic electronic configuration on the metal site avoiding potential paramagnetic NMR effects caused by the metal center.^31,32^ The syntheses in this work were based on that previously reported by Sheberla et al., and in each case, successful synthesis of crystalline Ni_3_(HITP)_2_ was confirmed by powder X-ray diffraction (PXRD), with inspection of the diffraction angle of the peaks indicating a consistent crystallographic pore size for all samples (SI Figure S1).^33^

The samples were subsequently characterized by 77 K N_2_ sorption isotherms (SI Figure S2), and despite a consistent synthetic procedure, a range of Brunauer–Emmett–Teller (BET)-specific surface areas (SSAs) were calculated for the six samples labeled A–F, ranging from 309 to 894 m^2^ g^–1^ (SI Figure S2 and SI Table S2). These values are consistent with the range previously reported for this MOF in the literature (260–885 m^2^ g^–1^).^5,32,34−42^ We note that even our best samples (samples A and F at 852 and 894 m^2^ g^–1^) have significantly lower BET SSAs than the reported theoretical value of 1370 m^2^ g^–1^ for this MOF, indicating a significant potential for pore blockages from impurities or sample defects.^43^ Scanning electron microscopy (SEM) of selected samples confirmed the expected rod-like morphology of the particles (SI Figure S3).^5,32,34,35,38−41^ Microanalysis highlighted some deviation from the expected stoichiometric quantities of elements, indicating varying levels of defects and impurities in the samples with the additional presence of some chlorine impurities from the starting materials, as previously reported by Sun et al. (SI Table S3).^38^ After characterizing the synthesized Ni_3_(HITP)_2_ samples for their crystallinity, porosity, microstructure, and chemical composition, the samples were employed in subsequent NMR studies. Unless otherwise specified, results reported below are from samples with a measured BET SSA within 5% of the highest reported BET SSA in the literature and thus considered to be the highest-quality samples.

### Investigation of Anionic Environments with NMR

**Figure 2:**
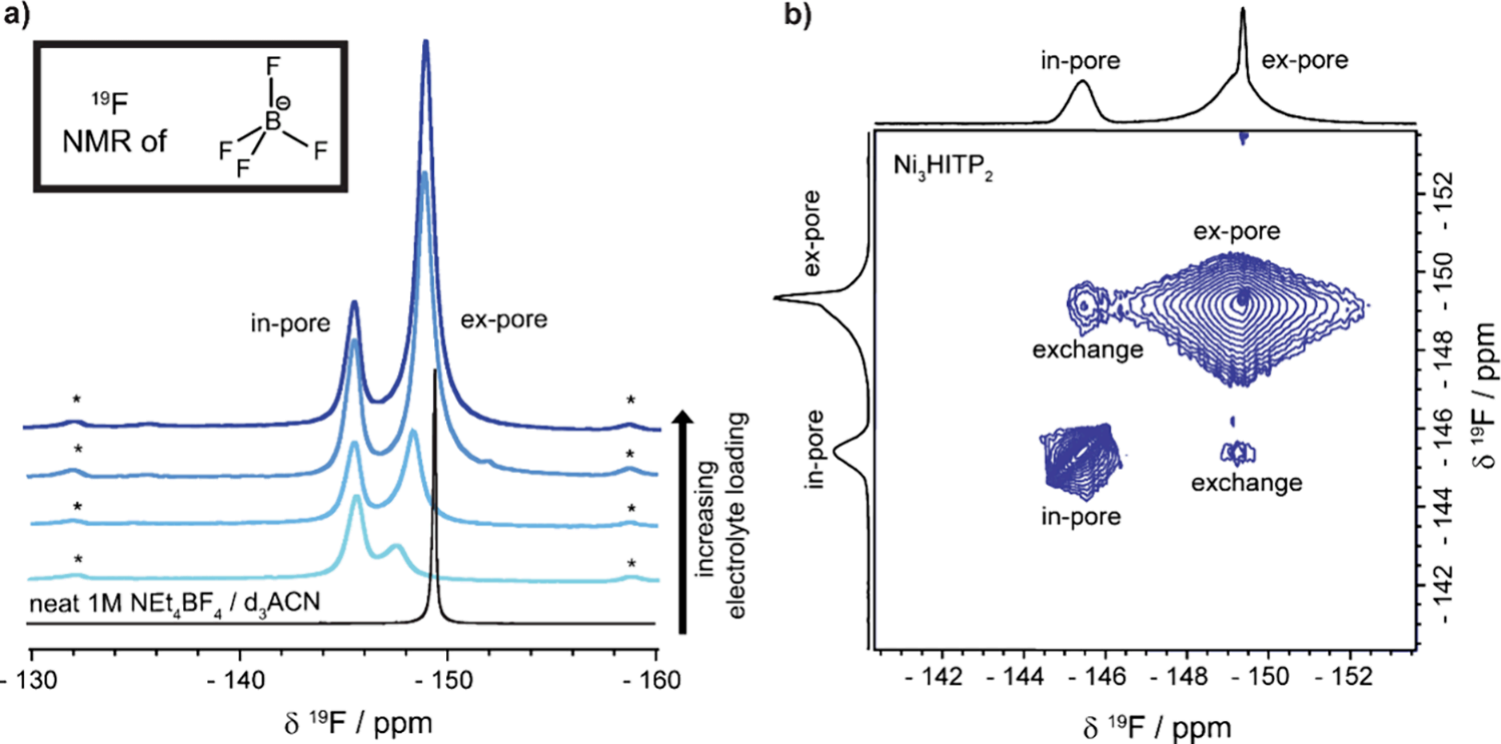
^19^F solid-state NMR (9.4 T) experiments at 5 kHz MAS. (a) Quantitative spectra of powder Ni_3_(HITP)_2_ sample A at various loadings of 1 M NEt_4_BF_4_/d_3_ACN electrolyte compared to the ^19^F NMR of the neat electrolyte. Spinning sidebands for the in-pore peak are denoted by *. The darkening shade of blue indicates progressively higher electrolyte loadings, from light blue to dark blue: 0.3, 0.5, 1.2, and 1.3 g of electrolyte per g of Ni_3_(HITP)_2_. (b) EXSY experiment with a mixing time of 50 ms showing chemical exchange between in-pore and ex-pore anions in a composite film of Ni_3_(HITP)_2_ sample A soaked with 1 M NEt_4_BF_4_/d_3_ACN electrolyte.

To study the electrolyte ion adsorption in Ni_3_(HITP)_2_ using NMR techniques, powdered MOF samples were combined with different loading volumes of 1 M tetraethylammonium tetrafluoroborate in deuterated acetonitrile (1 M NEt_4_BF_4_/d_3_ACN) electrolyte and ^19^F NMR spectra were recorded to investigate the BF_4_^–^ anion environments. (Figure 2a). Each ^19^F NMR spectrum revealed two resonances, indicative of two major BF_4_^–^ anion environments in the system, which can be initially assigned to “in-pore” and “ex-pore” environments (SI Figure S4a). The peak at approximately −145.5 ppm is assigned to in-pore anions as it is shifted by a greater extent away from the neat electrolyte due to MOF–anion interactions, while the peak at approximately −148.5 ppm is much closer to the neat electrolyte’s chemical shift and is assigned to ex-pore BF_4_^–^ species. The in-pore peak is additionally identified by more intense magic-angle spinning (MAS) sidebands, which from fitting various spectra of various samples with a chemical shift anisotropy (CSA) model (SI Table S4) consistently indicated a greater anisotropy experienced in this confined electrolyte environment compared to the more mobile ex-pore electrolyte despite variation in the absolute CSA value between samples.

Our peak assignments were further supported by integration of the NMR spectra at variable loadings (SI Figure S5a). At the lowest loading, the in-pore environment dominates, but upon increasing the solvent loading, this peak grows slowly compared to the proposed ex-pore environment, which dominates at higher electrolyte loadings where the in-pore environment, i.e., the electrolyte-accessible porosity of the MOF, becomes saturated. A series of analogous adsorption experiments on Ni_3_(HITP)_2_ composite electrode film gave rise to the same two environments, following the same trend on variation of electrolyte loading (SI Figure S6).

Having made these initial assignments, the ion dynamics of the system were investigated. Interestingly, the peak assigned as “ex-pore” shifts toward the neat electrolyte peak at increasing electrolyte loading (Figure 2a and SI Figure S5b). This change in chemical shift could not be accounted for purely by those expected for variation in the local electrolyte concentration (SI Figure S7), which suggests that this peak is impacted by fast exchange on the NMR time scale between truly unconfined electrolyte in the “ex-pore” environment and a smaller proportion of electrolyte, which is influenced by the MOF, likely close to the pore openings such that it is easily accessible for exchange. Exchange spectroscopy (EXSY) was used to further reveal the slow exchange between in-pore and ex-pore environments, which manifests as cross peaks appearing on a time scale of tens of milliseconds (Figure 2b). Despite these various exchange contributions, the adsorption experiments on Ni_3_(HITP)_2_ confirmed the presence of two key environments, in-pore and ex-pore, analogous to those seen in adsorption NMR studies on activated porous carbons.^19^

**Figure 3:**
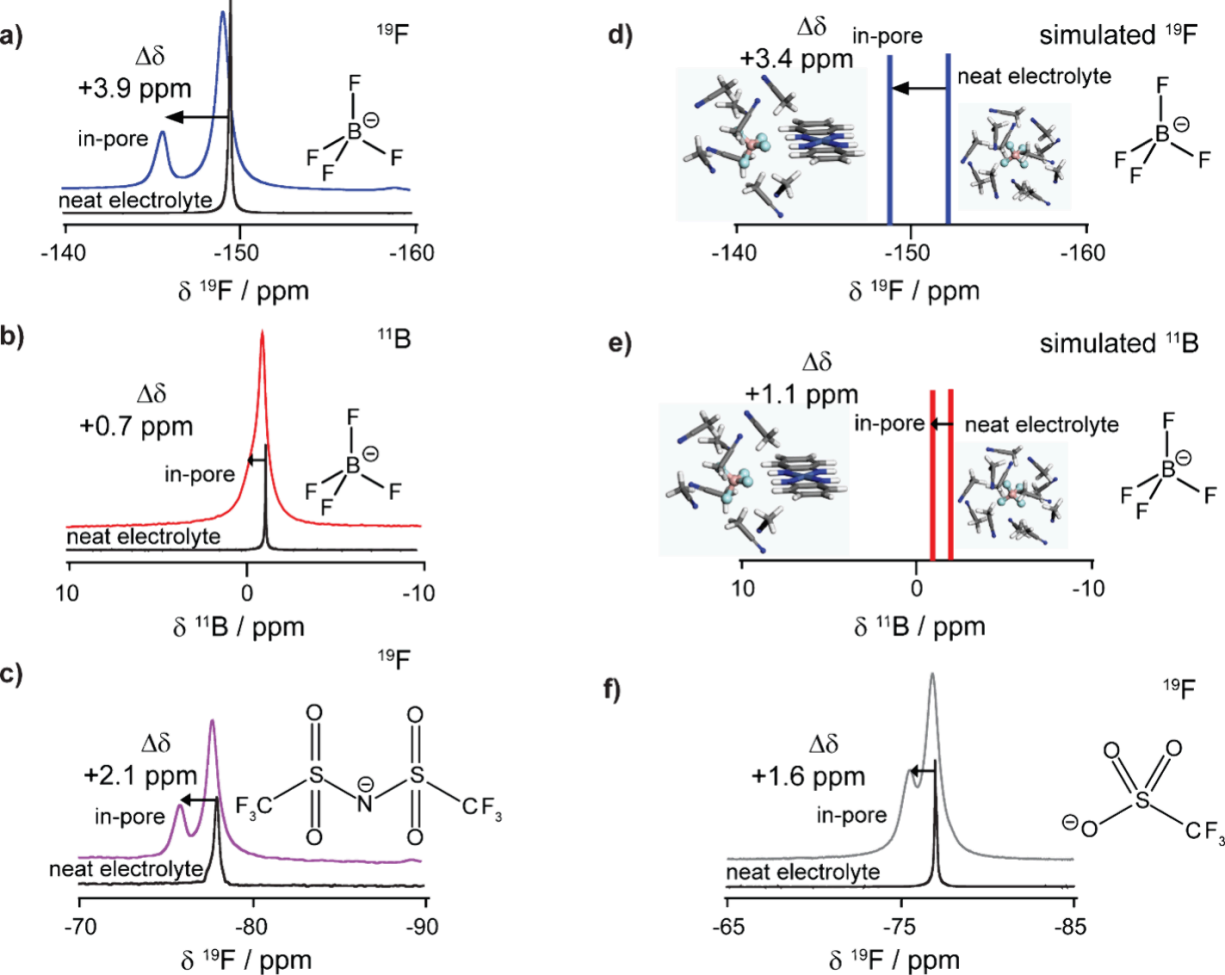
(a) ^19^F and (b) ^11^B solid-state NMR (9.4 T) spectra of Ni_3_(HITP)_2_ soaked with 1 M NEt_4_BF_4_/d_3_ACN compared to neat electrolyte. (c) ^19^F solid-state NMR (9.4 T) spectra of Ni_3_(HITP)_2_ soaked with 1 M NEt_4_TFSI/d_3_ACN compared to neat electrolyte. (d) ^19^F and (e) ^11^B simulated NMR spectra of tetrafluoroborate anion close to the Ni_3_(HITP)_2_ MOF fragment compared to in bulk acetonitrile. Spectra are schematic to show the simulated chemical shift; the line width is not indicative of the peak width. (f) ^19^F solid-state NMR (9.4 T) spectra of Ni_3_(HITP)_2_ soaked with 1 M NaSO_3_CF_3_/DMF compared to neat electrolyte. All experimental spectra used Ni_3_(HITP)_2_ sample A and are recorded in the quantitative regime with an MAS rate of 5 kHz.

Interestingly, the “in-pore” BF_4_^–^ peak for Ni_3_(HITP)_2_ is positively shifted from the neat electrolyte resonance, quantified by Δδ = δ_in-pore_ – δ_neat_ = +3.9 ppm (Figure 3a). This contrasts with the negative Δδ values seen in NMR studies of ion adsorption in porous carbons, where ring-current shielding effects dominate the Δδ values, leading to a NICS.^19^ As a result, in porous carbons, the observed Δδ remains constant when varying the NMR active nucleus studied or the electrolyte components. In MOFs, other noncovalent binding interactions may dominate the chemical shifts, with one possibility in Ni_3_(HITP)_2_ being hydrogen-bond interactions between the BF_4_^–^ anions and the N–H moiety of the HITP linker.^20,26^

To investigate the origin of the observed Δδ values in Ni_3_(HITP)_2_, both the studied NMR active nucleus and the electrolyte anion were independently varied (Figure 3 and SI Table S5). Studying the same sample in the original electrolyte of 1 M NEt_4_BF_4_/d_3_ACN with ^11^B NMR as a second probe of BF_4_^–^, the two peaks are no longer well-resolved (Figure 3b). On deconvolution, Δδ for the ^11^B spectrum was found to be just +0.7 ppm (SI Table S5). The significant difference in the ^19^F and ^11^B Δδ values for Ni_3_(HITP)_2_ is in contrast to previous findings on porous carbons.^19^ Indeed, previous work on a microporous carbide-derived-carbon showed very similar ^19^F and ^11^B Δδ values of −5.5 and −5.7 ppm, respectively.^19^ These shifts are dominated by a ring-current shift, which is assumed to be similar on average for both nuclei due to the rapid rotation of the BF_4_^–^ anion in the electrolyte.^20^ Therefore, the observed nucleus-*dependent* effects in Ni_3_(HITP)_2_ suggest a different dominant chemical shift mechanism compared with porous carbons.

To continue exploring the origin of the Δδ values in Ni_3_(HITP)_2_, the ion investigated via NMR spectroscopy was varied. Preliminary data showed poor resolution in ^1^H NMR spectra, suggesting a small Δδ and making identification and accurate quantification of NEt_4_^+^ cation environments difficult (SI Figure S8). Furthermore, ^19^F NMR was used to investigate the adsorption environments for TFSI^–^ anions in Ni_3_(HITP)_2_ in 1 M tetraethylammonium bis(trifluoromethylsulfonyl)imide (NEt_4_TFSI) in an acetonitrile electrolyte. The same two anion environments were evident in the spectrum, but with a measured Δδ for the “in-pore” environment of +2.1 ppm (Figure 3c and SI Table S5). Hence, these nucleus- and anion-*dependent* results therefore demonstrate that ring-current effects, while perhaps a contributor, are not dominant for this system, leading to a hypothesis that noncovalent interactions dominate the observed shifts. We further propose that the Δδ value is related to the strength of the interaction between the anions and the MOF functionality. DFT calculations confirmed a larger charge density on the fluorine atoms for the BF_4_^–^ anion compared to the TFSI^–^ anion as expected, leading to a stronger specific interaction alongside a larger observed Δδ. Additionally,^11^B NMR above gave rise to a low Δδ of just +0.7 ppm as boron, unlike fluorine, is not directly participating in a specific interaction with the MOF pore wall. These experimental results therefore support the hypothesis that the MOF–electrolyte interaction strength is modulated by the charge density on the fluorine (SI Figure S9).

To further investigate the proposed specific MOF–electrolyte interactions, chemical shift calculations were carried out on structural fragments extracted from hybrid QM/MM simulations of the Ni_3_(HITP)_2_–NEt_4_BF_4_/ACN system (see SI and Figure S10 for details). The simulations predicted respective ^19^F and ^11^B NMR Δδ values of +3.4 and +1.1 ppm calculated for a BF_4_^–^ anion in close proximity to a MOF fragment, compared to an anion in bulk acetonitrile (Figure 3d,e and SI Table S5), in close agreement with the experimental values. Furthermore, simulations in the absence of an applied electrochemical potential show that the anions are distributed over a range of sites within the pore but overall favor sites close to the MOF pore walls, adjacent to the N–H groups (SI Figure S11). Collectively, these results support the hypothesis that the ^19^F and ^11^B chemical shifts arise predominantly from a hydrogen-bond type interaction of the fluorine atoms with the hydrogens in the MOF N–H groups and that the value of Δδ may be linked to the strength of that interaction. It is these specific interactions that are responsible for the high resolution of the in-pore environment in the ^19^F NMR. Thus, anions closer to the center of the MOF pore would have a lower Δδ if measured directly, but as they are in fast exchange with the anions on the edge of the pores, the observed Δδ is a weighted average of all the anion environments. This specific anion-MOF specific interaction may also explain why the same effect is not seen in the ^1^H NMR spectra of the electrolyte cations, which will not undergo the same favorable hydrogen-bonding interaction with the MOF (SI Figure S8).

With the opportunity to study this MOF–electrolyte anion interaction, we further employed NMR to study a system previously reported to have negligible ion uptake (at null potential), by soaking Ni_3_(HITP)_2_ with 1 M sodium triflate in dimethylformamide (NaSO_3_CF_3_/DMF) electrolyte (Figure 3f).^14^ The resulting spectrum closely resembles those of the other organic electrolyte systems, with two peaks that we assign to in-pore and ex-pore environments, and a ^19^F Δδ of +1.6 ppm. The charge density on the fluorine atoms in the SO_3_CF_3_^–^ anion from DFT calculations is similar to that of the TFSI^–^ anion, supporting a similarly weak interaction with the MOF compared to BF_4_^–^ (Figure 3a) and thus a smaller Δδ (SI Figure S9). As before, note that significant spinning sidebands are present only for the more anisotropic in-pore environment in this system, supporting our assignments (SI Figure S12 and SI Table S4). Overall, our results suggest that there is in fact a significant in-pore anion population even without charging, in contrast with the negligible ion uptake previously reported by He et al. These findings highlight the significant power of ^19^F NMR to probe ion adsorption and specific MOF–electrolyte interactions.

### Correlating In-Pore Anion Population in the Absence of Applied Potential to Electrochemical Performance

**Figure 4:**
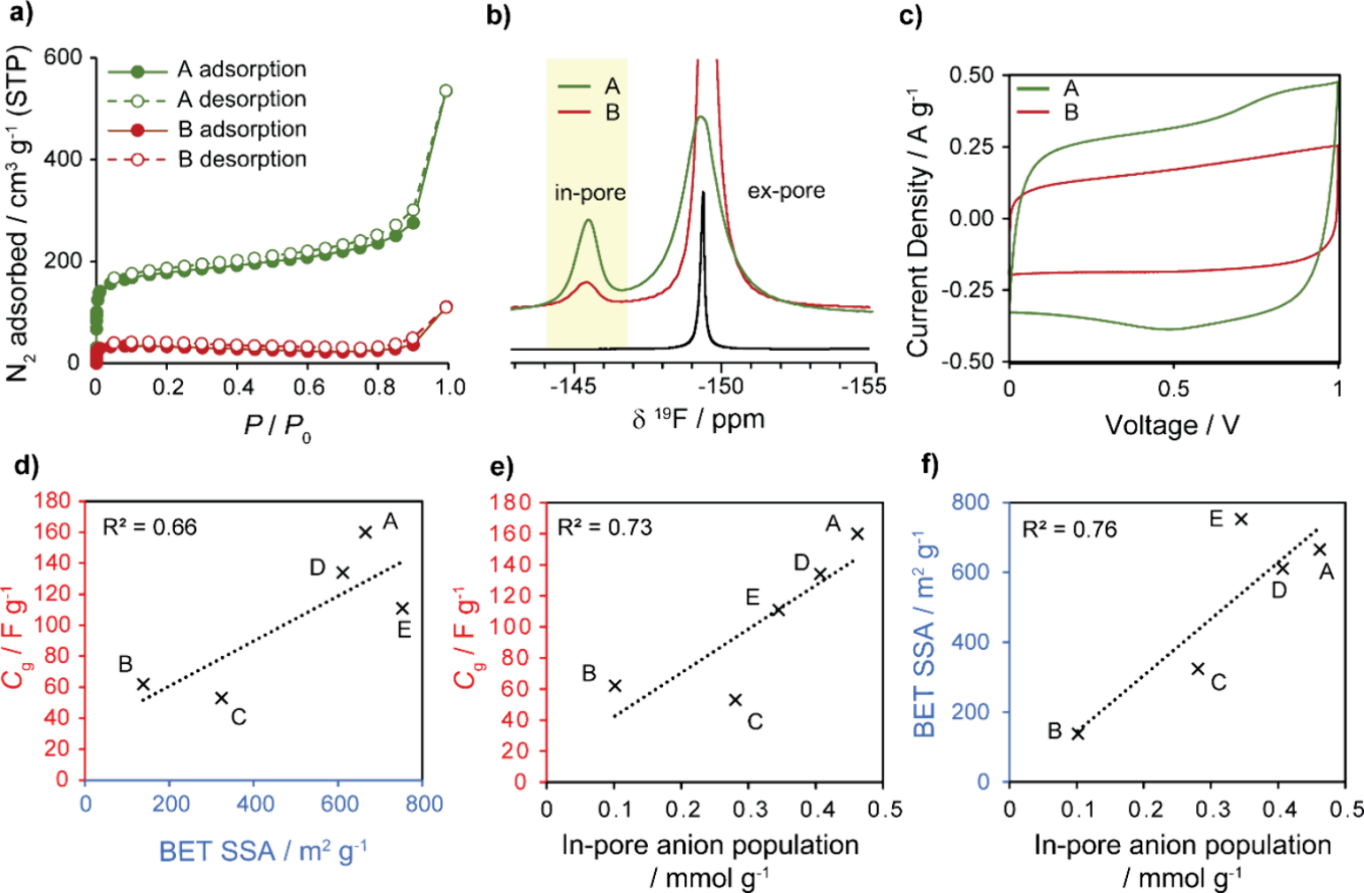
(a) N_2_ gas sorption isotherms at 77 K of Ni_3_(HITP)_2_ composite films of sample A (high BET SSA) and B (low BET SSA). (b) Quantitative ^19^F solid-state NMR (9.4 T) spectra at 5 kHz MAS of Ni_3_(HITP)_2_ composite films of samples A and B at saturated electrolyte loading volumes of 1 M NEt_4_BF_4_/d_3_ACN, compared to a neat electrolyte spectrum; differences in intensity of the in-pore peak are highlighted. (c) CVs at 5 mV s^–1^ of symmetric supercapacitors with Ni_3_(HITP)_2_ samples A and B as the active electrode material. The small faradaic contribution prominent in the CV for sample A is attributed to the previously reported quasi-reversible oxidation process of the MOF, reported to be likely centered on the linker molecule.^5^ Correlations between (d) BET SSA and gravimetric capacitance, (e) in-pore anion population and gravimetric capacitance, and (f) in-pore anion population and BET SSA for the five Ni_3_(HITP)_2_ composite film samples studied. All samples reflect a single gas sorption measurement except sample E, which is represented as an average of two measurements. Gravimetric capacitances at a current/density 0.05 A g^–1^ were measured from galvanostatic-charge–discharge (GCD) experiments.

^19^F NMR adsorption experiments, analogous to those previously described, were subsequently evaluated as a tool to predict the electrochemical performance of the Ni_3_(HITP)_2_ samples. Two samples with contrasting BET SSAs, sample A at 852 m^2^ g^–1^, and sample B at 309 m^2^ g^–1^, were selected and made into composite electrode films for further BET measurements (Figure 4a), NMR adsorption experiments (Figure 4b), and electrochemical performance tests (Figure 4c and SI Table S2). Adsorption experiments were performed at high electrolyte loadings to saturate the porosity of the MOF and probe the electrolyte-accessible in-pore volume (SI Table S2). Sample A was found to have a higher in-pore anion population (defined by millimolar in-pore anions per gram of MOF), evident from the larger integral of the in-pore peak in adsorption experiments (Figure 4b). Furthermore, we note a difference in the peak shape of the ex-pore environments between the two samples, which we attribute to differences in ion exchange rates.

Importantly, sample A demonstrated a higher gravimetric capacitance measured from galvanostatic charge–discharge (GCD) experiments (SI Table S2), which was also supported by a larger cyclic voltammogram (CV) area for a symmetric supercapacitor cell (Figure 4c). The CVs for both samples over a cell voltage window of 1.0 V qualitatively resemble the electric double-layer behavior previously reported for this system.^5,41^ However, with a gravimetric capacitance of 160 F g^–1^ at a current density of 0.05 A g^–1^ (0.3 mA cm^–2^), sample A exceeds the electrochemical performance reported in the literature for the same system (up to 111 F g^–1^ at a current density of 0.05 A g^–1^) and competes closely with the gravimetric capacitance of Ni_3_(HITP)_2_ with aqueous sodium sulfate electrolyte (170 F g^–1^ at a current density of 0.1 mA cm^–2^) reported by Nguyen et al.^5,41,44^ With a gravimetric capacitance of 62 F g^–1^, sample B falls within the range reported by Borysiewicz et al.^41^ The batch-to-batch variation observed in electrochemical performance may be linked to variations in MOF morphology, as has previously been reported for Ni_3_(HITP)_2_.^41^ Morphology differences would also be expected to give rise to changes in exchange rate between the in-pore and ex-pore environments, which would explain the significant difference in peak shape of the ex-pore environment.

To assess in more detail how well NMR adsorption experiments in the absence of an applied potential predict electrochemical performance, gravimetric capacitance was used (SI Table S2), which was plotted as a function of both the BET SSA (Figure 4d and SI Figure S13) and in-pore anion population obtained by NMR for composite films of five samples of Ni_3_(HITP)_2_ samples (A–E; Figures 4e and SI Figure S14). We observe that the NMR characterization provides a rough indication of gravimetric capacitance through the correlation of the two properties (*R*^2^ = 0.73) (Figure 4e). For our samples, we found adsorption NMR to predict gravimetric capacitance to an accuracy similar to that of BET SSA (*R*^2^ = 0.66), which is conventionally used to screen conductive MOF quality (Figure 4d). As such, there is also some correlation between in-pore ion population and BET SSA itself (*R*^2^ = 0.76), highlighting the ability of NMR to effectively probe porosity of layered MOFs (Figure 4f). However, since our NMR experiments probe electrolyte-accessible pore volume, whereas the BET analysis is derived from N_2_ gas sorption data, we anticipate some deviation between the correlations. This is highlighted by the unexpectedly high BET SSA for sample E, given its associated gravimetric capacitance (Figure 4d) and in-pore anion adsorption (Figure 4f). Nevertheless, we see that the adsorption NMR experiments still accurately reflect the gravimetric energy storage performance in this sample (Figure 4e). Due to the anomalously high BET SSA of the composite film of sample E, the in-pore anion population was also plotted with the BET SSA of the powder MOF samples to further probe the relationship between NMR adsorption and gas sorption experiments (SI Figure S15). This showed an improved correlation (*R*^2^ = 0.99), supporting our initial results. Importantly, as our ^19^F NMR experiments take only minutes to acquire compared to hours/days for gas sorption measurements, this work shows that adsorption NMR experiments could be used as an alternative to gas sorption where higher throughput screening of MOF samples is required (Figure 4e,f).

### *Ex-Situ* NMR Charging Experiments

**Figure 5:**
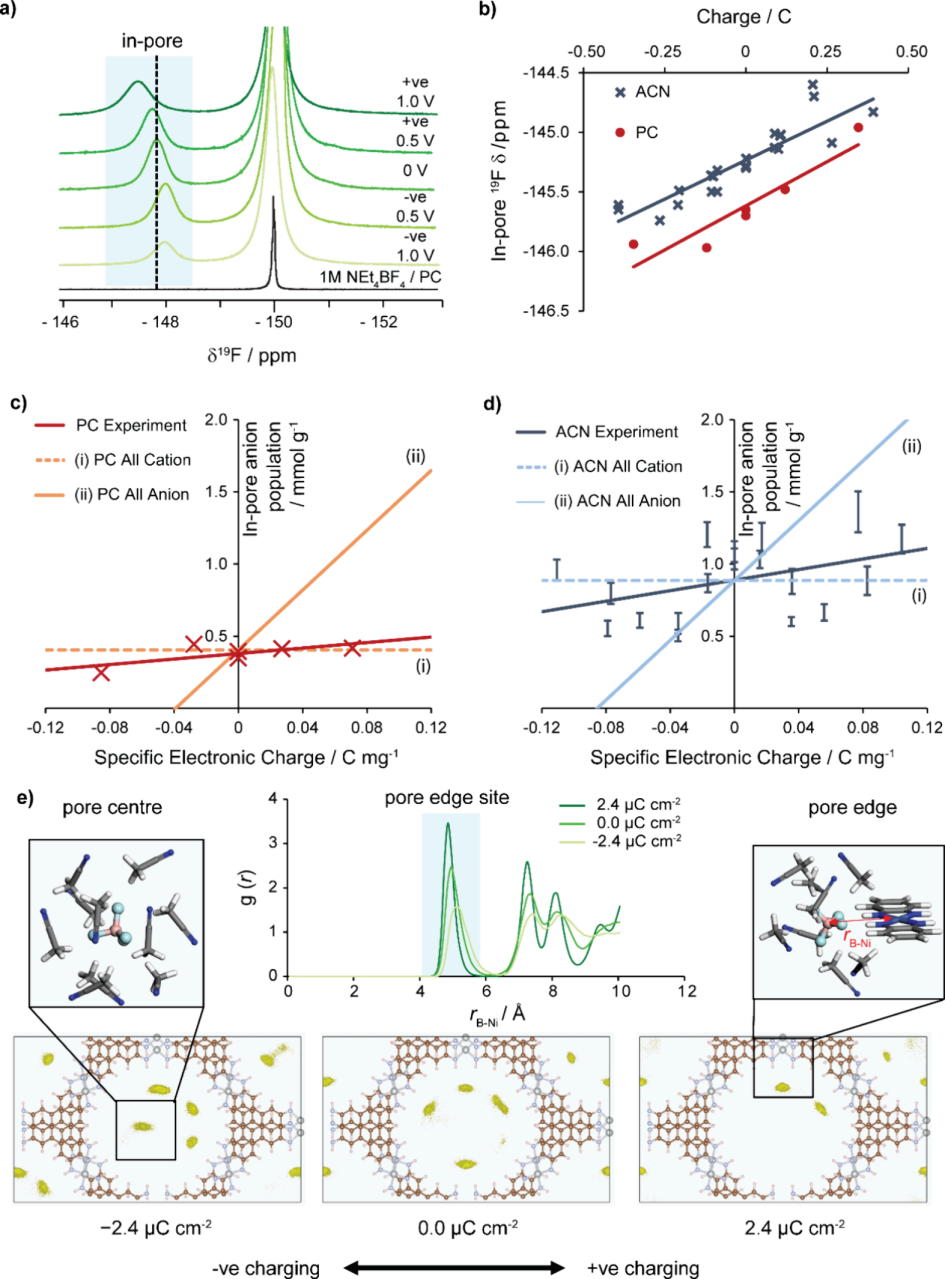
Studies on charging Ni_3_(HITP)_2_ supercapacitor electrodes with organic electrolytes. In the *ex-situ* experiments, symmetric supercapacitors were held at a constant voltage for 1 h as the current was monitored, and then disassembled, with NMR performed separately on each of the electrodes after disassembly. (a) ^19^F solid-state NMR (9.4 T) spectra at 25 kHz MAS of *ex-situ* electrodes of Ni_3_(HITP)_2_ with 1 M NEt_4_BF_4_ in propylene carbonate (PC) compared to neat electrolyte; to obtain peak resolution for accurate fitting, a higher MAS rate of 25 kHz was required for samples with propylene carbonate solvents (SI Figure S16d). (b) Correlation between in-pore chemical shift and electrode charge for 1 M NEt_4_BF_4_ in deuterated acetonitrile (ACN) and propylene carbonate (PC). Measured in-pore ion population against specific electronic charge compared to theoretical scenarios in which all electrode charge storage is accounted for by exclusively (i) cation or (ii) anion movement, with intercept fixed at the same value as the regression line for ease of comparison of their gradients, for an electrolyte of 1 M NEt_4_BF_4_ in (c) propylene carbonate (PC) and (d) deuterated acetonitrile (ACN). (e) Top middle: radial distribution function, *g*(*r*), as a function of distance between the boron atom of BF_4_^–^ and nickel in Ni_3_(HITP)_2_, shows that the peak in the highlighted region corresponds to the pore-edge environment. Bottom row: QM/MM of simulated anion populations, highlighted in yellow, for the 1 M NEt_4_BF_4_ electrolyte in Ni_3_(HITP)_2_, under negative charging (left), zero charge (middle), and positive charging (right). The isosurface level is 0.003 e bohr^–3^. Simulations follow the cation-dominated charging mechanism observed experimentally. Simulated anion population symmetry is not fully maintained in the 5 ns sampling time due to its strong binding affinity to the electrode. Two key anion sites are identified as pore center (top left) and pore edge (top right).

To explore the capacitive charging mechanism of Ni_3_(HITP)_2_, this MOF was employed as an active electrode material in *ex-situ* supercapacitor experiments, for which NMR is performed after charging and disassembly of the supercapacitor (Figure 5). *In-situ* NMR measurements, performed in real time on a charging supercapacitor, were not possible due to a lack of spectral resolution in the absence of MAS (SI Figure S16a). *Ex-situ*^19^F NMR spectra for all the electrodes showed the two expected resonances, corresponding to in-pore and ex-pore BF_4_^–^ anion environments (Figure 5a). Interestingly, a systematic shift of the in-pore resonance was also observed as the charging voltage was varied (Figure 5b). The experiments were conducted with 1 M NEt_4_BF_4_ in propylene carbonate (PC), a less volatile solvent, in addition to the original 1 M NEt_4_BF_4_ in an acetonitrile electrolyte (SI Figure S16b). Similar results were obtained for the two electrolyte systems despite concerns about solvent evaporation with the acetonitrile-based electrolyte (SI Figure S16c).

Through using calibration experiments, quantitative in-pore BF_4_^–^ populations were obtained at different charging voltages (SI Figure S17). For both the PC (Figure 5c), and the acetonitrile electrolytes (Figure 5d), the in-pore anion population was plotted against the specific electronic charge of the electrode alongside different theoretical scenarios. The first is (i) an “all cation” scenario in which the cations account for all of the electrode’s charge storage and there is no net change in the in-pore anion population, i.e., charge is stored through cation adsorption (counterion adsorption) for the negative electrode, and through cation desorption (co-ion desorption) for the positive electrode. The second case is (ii) an “all anion” scenario in which the anions account for all the electrode’s charge storage. Both cases are for illustration of the gradient only, and the intercept is fixed to match that of the experimental regression slope. By comparison of the experimental slope from regression with the theoretical scenarios, the anions accounted for 9 ± 13% of the total charge storage in a PC electrolyte, and 18 ± 20% in an acetonitrile electrolyte (95% confidence interval, see the SI for details). Therefore, the experimental result for both electrolytes is closer to the “all cation” scenario. This gives confidence that regardless of the organic solvent, cations are the major contributor to charge storage in Ni_3_(HITP)_2_. This finding supports the cation-dominated charging mechanism proposed from theoretical and experimental work for the related MOF Cu_3_(HHTP)_2_.^12,13^ Therefore, while in-pore anion population was earlier shown to serve as a useful indicator of electrolyte-accessible pore volume and hence electrochemical performance (Figure 4e), the anions themselves are not primarily responsible for the charge storage in these systems.

Despite a relatively small change in the total in-pore anion population on charging, it was possible to detect more subtle charging processes by monitoring the chemical shift of the in-pore environment. It was found that the in-pore chemical shift in both electrolytes was strongly correlated with the charge stored in the electrode, giving rise to a linear relationship (Figure 5b). We propose that this variation of the Δδ value is related to changes in both the distribution of anions between pore-center and pore-edge sites (which are expected to be in fast exchange on the NMR time scale) and the strength of the specific interaction between the anion and the MOF N–H sites. Further QM/MM simulations with charging were used to investigate this behavior (Figure 5e and SI Table S3). On negative charging (Figure 5e, bottom left), a dramatic change in anion distribution is observed relative to the uncharged scenario (Figure 5e, bottom middle), with a significant shift in anions toward the pore center. This can be attributed to the now less favorable interactions with the net negative MOF pore edge. The drop in anion population at the pore-edge site is indicated by the reduced integral of the peak around 5 Å in the radial distribution function (Figure 5e, top middle). Any anions remaining close to the pore edge will also experience a weaker H-bonding interaction owing to a smaller partial positive charge on the H atom of the MOF N–H groups, and this is amplified by a shift of the pore-edge environment away from the MOF walls also seen in the radial distribution function, with the peak around 5 Å shifting to a greater *r*_B–Ni_ distance (Figure 5e, top middle). These synergistic effects lead to a reduced Δδ value during negative charging. Conversely, on positive charging (Figure 5e, bottom right), the anions interact more favorably with the edge of the MOF, where the N–H groups become more positively charged, and so anions migrate toward the pore boundary of the MOF, resulting in a higher pore-edge population in closer proximity to the N–H groups (Figure 5e, top middle). Therefore, an increase in the average Δδ value is observed. A similar behavior of anion rearrangements has previously been proposed in the related MOF, Cu_3_(HHTP)_2_.^12^ These experimental observations were found to be in qualitative agreement with calculated ^19^F chemical shift values from charging of aMOF fragment using implicit solvation of an adsorbed BF_4_^–^ anion (SI Figure S18). Therefore, while the total in-pore anion population is almost invariant as cations dominate the charge storage for these conductive layered MOF systems, NMR has the power to track subtle changes in the anion distribution within the pore structure and the strength of the MOF–electrolyte interaction.

### *Operando* EQCM Experiments

**Figure 6:**
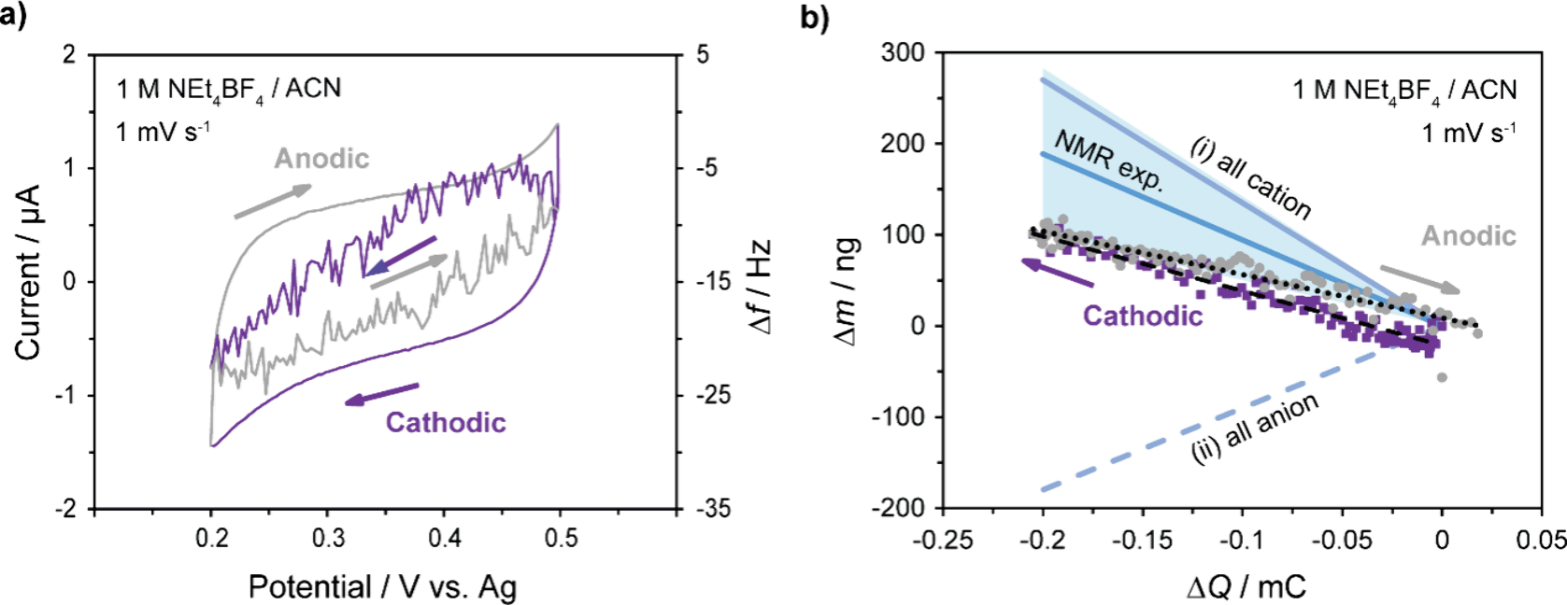
(a) CV (outside lines) and EQCM frequency response (inside lines) of Ni_3_(HITP)_2_ with 1 M NEt_4_BF_4_/ACN electrolyte, at a scan rate of 1 mV s^–1^. The potential range was restricted to +200 to +500 mV vs Ag to avoid the possible decomposition reactions at the MOF/gold electrode of the EQCM cell. (b) Plot of experimental electrode mass change, Δ*m*, calculated according to Sauerbrey’s equation from the frequency response, Δ*f*, shown in (a), against accumulated charge (Δ*Q*). Δ*Q* was calculated by integrating the current against time for the CVs and setting the charge at the electrode potential of +500 mV vs Ag to zero. Δ*f* and Δ*m* are considered separately for cathodic (purple) and anodic (gray) polarizations. The average mass change is given by the black dashed line (cathodic scan) and the black dotted line (anodic scan). These experimental results are compared with theoretical scenarios, in which all electrode charge storage is accounted for by exclusively (i) cation or (ii) anion adsorption, and the predicted range of Δ*m* calculated from *ex-situ* NMR experiments with an 18 ± 20% anion contribution indicated by the shaded blue region. All of these scenarios exclude any contributions from movement of solvent.

As ^1^H NMR could not accurately quantify the environments of the cations due to poor resolution of the proton spectra (SI Figure S7), EQCM experiments were performed with Ni_3_(HITP)_2_ with 1 M NEt_4_BF_4_ in acetonitrile electrolyte to further investigate the charging mechanism (Figure 6). In EQCM, the frequency change, Δ*f*, of a crystal is measured and used to determine the change in mass, Δ*m,* of an electrode during charging via Sauerbrey’s equation, as long as the gravimetric approach is valid (see the SI for details, SI Figure S19).^45^ This change in mass can be attributed to movement of cations, anions, and solvent into and out of the electrode. To prevent possible decomposition reactions that occur on the MOF/Au electrode of the EQCM cell, the potential window was restricted to +200 to +500 mV vs Ag during the EQCM experiment (Figure 6a; CVs shown in outside lines with frequency change (Δ*f*) shown inside). Given that the open circuit potential of the electrode, prior to any polarization, was +284 mV relative to Ag, the selected potential window covers in part both positive- and negative-charging regimes of the *ex-situ* NMR experiments above. Furthermore, by using a slow voltage sweep of 1 mV s^–1^, we assume that the mechanistic charging processes observed via EQCM can be directly compared to the results of the earlier *ex-situ* NMR experiments, which used constant voltage holds.

A reversible and negative correlation between Δ*m* and Δ*Q* was found during cathodic polarization (Figure 6b), which by comparison with the (i) all-cation and (ii) all-anion scenarios described above indicates that cations are the dominant charge carriers in this system.^45^ Therefore, the mass increase with negative electronic charge accumulation indicates net cation adsorption in the cathodic charging process, while a reversible net cation desorption process is observed in the anodic regime. Importantly, this agrees qualitatively with the *ex-situ* NMR results presented above and confirms the dominant contribution of the cations to the charging mechanism. The slope of the EQCM data for both cathodic and anodic charging corresponds to a relatively small change in mass on charging relative to the range expected from the experimental *ex-situ* NMR data (Figure 6b). This result suggests that, in addition to the dominant cation adsorption during cathodic charging, there must be a net loss of solvent and or/anion desorption, and vice versa on anodic charging. As only the net mass change is recorded in EQCM experiments, it is difficult to accurately quantify the absolute solvent and/or anion movement as molecules may simultaneously be moving into and out of the pores during both charging and discharging. To investigate the solvent contribution to the charging mechanism further, ^2^D NMR of the deuterated acetonitrile solvent in 1 M NEt_4_BF_4_/d_3_ACN was attempted, with the aim to quantify the “in-pore” solvent environment (SI Figure S20). However, due to poor resolution, fitting these spectra is associated with high uncertainty and, coupled with partial evaporation of acetonitrile in *ex-situ* measurements, makes the application of NMR limited in studying the solvent in this system. Nevertheless, this works highlights the complementary nature of EQCM together with NMR to study anion, cation, and solvent contributions to the charging mechanism of layered MOFs in supercapacitor devices. Qualitatively, a cation-dominated charging mechanism is observed in both NMR and EQCM charging experiments for this MOF system, although measuring the quantitative agreement in the percentage contribution of anion movement to charge storage is not possible.

Interestingly, the cation-dominated nature of this charging mechanism is consistent with EQCM studies on the related MOF Cu_3_(HHTP)_2_ with 1 M NEt_4_BF_4_/ACN electrolyte.^13^ The greater contribution of the cations compared to the anions in charge storage of these systems suggests that the cation identity may be most closely linked to the overall electrochemical performance. Gittins et al. previously demonstrated the impact of increasing the alkyl chain length in this family of cations, but further exploration of electrolytes is needed to improve electrochemical performance beyond that for 1 M NEt_4_BF_4_/ACN electrolyte. Conversely, this work may suggest that the cations are only the dominant contribution to charge storage in this system due to relatively strong BF_4_^–^ anion–MOF interactions. If the relative strengths of the interactions between the anions and the cations with the MOF could be systematically modified, then this could be exploited to control the extents to which each ion contributes to charge storage. Controlling the charging mechanism in this way would likely also give rise to a higher capacitive performance.

## Conclusions

This work has demonstrated the application of solid-state NMR spectroscopy for understanding the ion adsorption and charge storage mechanisms of conductive layered MOFs. Importantly, our NMR spectra of Ni_3_(HITP)_2_ soaked with a traditional supercapacitor organic electrolyte reveal distinct resonances for in-pore and ex-pore BF_4_^–^ anions, providing the opportunity to study the electric double layer in these materials during charging. The chemical shift of the in-pore resonance was further revealed to be determined by specific anion–MOF interactions, an observation that was supported by QM/MM and DFT simulations. Solid-state NMR has also demonstrated potential for high-throughput screening of the electrochemical performance of layered MOFs, with significantly improved efficiency compared to traditional N_2_ gas sorption experiments. Furthermore, for the first time, *ex-situ* NMR measurements were used to gain experimental insights into the charging mechanism of a layered MOF, Ni_3_(HITP)_2_, with organic electrolytes, and revealed only a minimal contribution of anions. Subtle changes in the in-pore chemical shift were seen on charging, which in combination with QM/MM simulations are linked to a redistribution of anions between two key in-pore environments. Given the coupled experimental challenges of obtaining peak resolution in NMR (i) under static conditions and (ii) of the cation environments in this system, we emphasize the need for both the development of spinning *in situ* NMR systems and combining NMR with other techniques for a complete mechanistic study.^47,48^ As such, EQCM experiments supported the idea that cations are the dominant charge carriers in this system and additionally highlighted the importance of the solvent in the charging mechanism, a contribution which is yet to be fully understood in these systems. The specific MOF–electrolyte interactions observed by NMR in these systems are in contrast with activated carbons typically used in supercapacitors and therefore present a unique opportunity for MOF-based systems. These interactions may be explored and exploited further to modify their strength, and therefore to both modulate the charging mechanism, and optimize supercapacitor performance. As such, this work offers a number of pathways for solid-state NMR to be used to aid in the future design of supercapacitors.

## Data Availability

All raw experimental data files are available in the Cambridge Research Repository, Apollo, with the identifier DOI: 10.17863/CAM.107715

## Supporting Information Available

Additional experimental and simulation details; methods and materials; detailed discussion of NMR and EQCM analyses; supporting figures including PXRD, SEM, and gas sorption isotherms; additional NMR spectra; and fitting and simulation results (PDF)
