# Supplementary material for: Revealing Ion Adsorption and Charging Mechanisms in Layered Metal–Organic Framework Supercapacitors with Solid-State Nuclear Magnetic Resonance
Source: J Am Chem Soc. 2024 Aug 12;146(33):23171–81. doi: 10.1021/jacs.4c05330 (PMC11345813; doi:10.1021/jacs.4c05330)
Supplement: Supplementary file 1 — ja4c05330_si_001.pdf [file ja4c05330_si_001.pdf]

Supplementary Information for:

Revealing Ion Adsorption and Charging Mechanisms in Layered Metal-Organic Framework Supercapacitors with Solid-State Nuclear Magnetic Resonance

Chloe J. Balhatchet[1], Jamie W. Gittins[1], Seung-Jae Shin[2], Kangkang Ge[3], Xinyu Liu[1], Teedhat Trisukhon[1], Shivani Sharma[1,4], Thomas Kress[1], Pierre-Louis Taberna[3,5], Patrice Simon[3,5], Aron Walsh[2], Alexander C. Forse[1*]

[1] Yusuf Hamied Department of Chemistry, University of Cambridge, Lensfield Road, Cambridge CB2 1EW, UK.

[2] Thomas Young Centre and Department of Materials, Imperial College London, London SW7 2AZ, UK.

[3] CIRIMAT, UMR CNRS 5085, Université Paul Sabatier Toulouse III, Toulouse 31062, France.

[4] Department of Chemical and Biomolecular Engineering and Department of Chemistry, University of California, Berkeley, California 94720, United States

[5] RS2E, Réseau Français sur le Stockage Electrochimique de l'Energie, FR CNRS, 3459, Amiens Cedex 80039, France.

[*]Corresponding author's email: acf50@cam.ac.uk

Table of Contents

Methods & Materials 1

Materials 1

Synthesis 1

Materials Characterisation 1

X-Ray Diffraction 1

Gas Sorption 1

Scanning Electron Microscopy 1

Elemental Analysis 1

Adsorption NMR Sample Preparation 1

NMR Experiments 2

DFT for Mulliken Population of Anions 2

QM/MM Simulation Details 2

Electrochemical Characterisation 3

Electrode Preparation 3

Supercapacitor Assembly 3

Electrochemical Cell Characterisation 3

Charging Mechanism Studies 4

Ex-Situ NMR Experiments 4

Ex-Situ NMR Analysis 4

Operando EQCM Experiments 4

Operando EQCM Analysis 4

SI Figure S1 6

SI Figure S2 7

SI Table S2 8

SI Figure S3 9

SI Table S3 10

SI Figure S4 11

SI Table S4 12

SI Figure S5 13

SI Figure S6 14

SI Figure S7 15

SI Table S5 16

SI Figure S8 17

SI Figure S9 18

SI Figure S10 19

SI Figure S11 20

SI Figure S12 21
SI Figure S13 22
SI Figure S14 23
SI Figure S15 24
SI Figure S16 25
SI Table S4 26
SI Figure S16 27
SI Figure S17 28
SI Figure S18 29
SI Figure S19 30
SI Figure S20 31
References 32

Methods & Materials

Materials

Chemical reagents were purchased from Sigma-Aldrich and used as commercially supplied and used without further modification unless otherwise specified: *N*,*N*-dimethylformamide (DMF), aqueous ammonia (NH_4OH, 35 % NH_3), and acetone from Fisher Scientific; 2,3,6,7,10,11-hexaiminotriphenylene hydrate ($H_6HITP.xH2O$) from Chemextensions; tetraethylammonium (NEt_4BF_4), tetramethylammonium bis (trifluoromethanesulfonyl)imide (NEt_4TFSI) and sodium trifluoromethanesulfonate (NaOTf) were dried under vacuum at 100 °C then transferred to an N_2-filled glovebox; anhydrous acetonitrile (ACN) and *N,N*-Dimethylformamide (DMF) were purged with N_2 for 3 h and transferred to an N_2-filled glovebox then further dried by the addition of activated 3 Å molecular sieves (activated under vacuum 250 °C, 12 h); anhydrous D_3-Acetonitrile (CD_3CN) vials were pre-rinsed and sealed under argon atmosphere then transferred to an N_2-filled glovebox.

Synthesis

The synthesis was carried out with minor modification from the published procedure of Sheberla *et al.*.[1]

A solution of 323 mg of $NiCl_2.6H_2O$ (1.36 mmol, 1.5 eq) in 20 mL of water was added to a solution of 487 mg (0.91 mmol, 1 eq) of HATP.6HCl in 140 mL of water. To this was added 3.5-4.5 mL of concentrated aqueous ammonia (18 M). The resulting mixture was heated to 60 ºC in an oil bath and stirred for 2 h with air bubbling through a needle. The resulting crude black precipitate was separated from the reaction mixture by centrifugation and washed with deionized water (8 x 135 mL) and ethanol (3 x 135 mL). Finally, the solid product was dried under dynamic vacuum on a Schlenk line at 85 ºC for a minimum of 48 h. The product was kept in a nitrogen-filled glovebox until used.

Materials Characterisation

X-Ray Diffraction

Powder X-Ray Diffraction data were obtained in-house using a Malvern Panalytical Empyrean instrument with non-chromated CuKα radiation (λ = 1.5406 Å) and a X'celerator Scientific detector. Samples were placed in a glass sample holder and measurements were performed at room temperature with a θ/2θ Bragg-Benato geometry. A divergence slit of 1/8 was found to be optimal, and a 2θ range of 3° to 50° with a step size of 2θ = 0.017° was used, with a resulting experiment time of 1 h. Simulated PXRD were produced using VESTA version 4.6.0.

Gas Sorption

An Anton Parr Autosorb iQ-XR was used to obtain all low pressure N_2 isotherms at 77 K. *Ex-situ* degassing (90-120 °C, 16 h) was performed for all samples according to Table S2. Degassing of the same sample at either side of this range of temperatures was not observed to give rise to a significant difference (<10%) in Brunauer–Emmett–Teller specific surface areas (BET SSA), and no batch-to-batch correlation was observed between degassing temperature and BET SSA.

AsiQwin version 5.21 software was used for evaluation of sorption isotherms and the calculation of BET areas using Rouquerol's consistency criteria.[2] All samples exhibited a Type I N_2 isotherm, indicative of microporosity in the MOF.

Scanning Electron Microscopy

$Ni_3(HITP)_2$ samples were mounted onto a stainless-steel scanning electron microscopy (SEM) stub. A Quorum Technologies Q150T ES Turbo-Pumped Sputter Coater was used to sputter-coat the samples with 10 nm Pt to improve the conductivity for imaging. Imaging was performed on a Tescan MIRA3 FEG-SEM, with a 5 keV beam voltage and working distance of 7 – 8 mm.

Elemental Analysis

Inductively Coupled Plasma Optical Emission Spectroscopy (ICP-OES) using a Thermo Scientific iCAP-7400 ICP spectrometer was used to determine Ni and Cl content. CHN combustion analysis (975 °C) using an Exeter Analytical CE-440 was used for for C, H, and N content.

Adsorption Sample Preparation for NMR Experiments

For adsorption sample preparation, activated $Ni_3(HITP)_2$ powder (Figures 2-3) or 85 wt. % composite films (Figure 4) were packed into zirconia Magic-Angle Spinning (MAS) rotors (2.5 mm outer diameter) in an N_2-glovebox. The rotor was weighed before and after adding the MOF material, then 1M NEt_4BF_4/d_3ACN electrolyte was added with a micro-syringe until the desired loading was reached and the capped rotor was weighed again. Samples were left overnight (> 16 h) for complete soaking before starting NMR experiments. Repeat NMR spectra on calibration samples demonstrated rotors were stable with respect to loss of electrolyte over a timescale of > 50 days, significantly longer than the typical over which NMR experiments were recorded (2-3 days).

Solid-State NMR Experiments

Solid-state NMR experiments were performed on a Bruker Avance Neo spectrometer with magnetic field strength of 9.4 T, corresponding to ^{19}F, ^{1}H and ^{11}B Larmor frequencies of 376.6 MHz, 400.2 MHz and 128.4 MHz. All NMR experiments were performed at room temperature using a Bruker 2.5 mm double-resonance MAS probe. All one-pulse (zg) spectra were recorded in the quantitative regime with respect to T_1 relaxation which is a condition for the peak area to linearly scale with the sample concentration. The spectra were recorded under magic angle spinning (MAS) which averages some anisotropic NMR interactions and sharpens NMR linewidths. As a compromise between improving the peak resolution and minimising the influence of electrolyte centrifugation of the spectrum, a relatively low MAS rate of 5 kHz was consistently used.[3,4]

T_1 relaxation measurements were performed using a saturation-recovery pulse sequence. The range of radiofrequency field (RF) strengths and quantitative recycle delays used for each nucleus is given in Table S1. The 90° pulse length was optimised on every sample.

Nucleus Studied	RF Strength / kHz	Quantitative Recycle Delay / s
^{19}F	58-119	4-16
^{1}H	139	8
^{11}B	89	4

Table S1: Range of RF strengths and quantitative recycle delays found for all NMR experiments in this work.

^{19}F chemical shifts were referenced externally to hexafluorobenzene at −164.9 ppm, ^{1}H to the ethanol -CH_3 environment at 1.2 ppm and ^{11}B to sodium borohydride at −42.1 ppm, all relative to tetramethylsilane (TMS). All spectra in this work are normalised according to the number of scans performed; spectra on the same figure are further normalised to the mass of MOF in the sample.

Fitting of spectra was performed using dmfit software using a Lorentzian line shape for all peaks and a chemical shift anisotropy model (Haeberlen convention). For adsorption experiments, in all cases two peaks representing in-pore and ex-pore environments were fitted (**SI Figure S4a**), and in some cases a third environment peak was used to represent free electrolyte. Fit parameters were not fixed between fits in order to obtain the best possible fits in each case and to avoid any bias as these are the first electrolyte adsorption NMR experiments performed on layered MOFs.

DFT simulations for Mulliken Population

Input xyz files for Gaussian calculations were generated on Avogadro, utilising the in-built forcefield geometry optimisation. Geometry optimisation was then performed using Gaussian 16 software using a range of functionals and basis sets as set out in Figure S9.[5] The solvent keyword was used to specify acetonitrile for the BF_4^- and $TFSI^-$ anions, and DMF for $SO_3CF_3^-$. As all fluorine atoms are chemically equivalent in the anions involved, average Mulliken populations were taken for each structure.

QM/MM Simulation Details

The mean-field QM/MM multiscale electrochemical simulation, namely, density functional theory in classical explicit solvents (DFT-CES)[6], is implemented in a bespoke code that combines the Quantum ESPRESSO[7] and LAMMPS[8]. The DFT-CES iteration was repeated until the difference of the DFT total energy between the iterations converged below 0.1 kcal mol^{-1}. At every iteration, 7 ns MD simulation is performed, and the last 5 ns trajectory is sampled to average the electrostatic potential of the electrolyte phase that was employed in the subsequent DFT calculation as an external potential.

The $Ni_3(HITP)_2$ electrode was quantum-mechanically modelled using a two-layer with a $(1 \times \sqrt{3})$ rect. unit cell with dimensions of 21.9 Å × 37.9 Å × 6.4 Å. The electrode has a slipped parallel AB stacking structure based on a previous study[9]. The Perdew–Burke–Ernzerhof (PBE) exchange-correlation functional with dispersion correction via Grimme's scheme (DFT+D3) was employed[10,11]. For an accurate description of the localised *d* electrons of Ni, the on-site Coulomb interaction was added to the *d* orbital of Ni with a U_d = 6.4 eV[12]. The projector-augmented-wave (PAW) method was used with a kinetic energy cutoff of 50 Ry[13], and the gaussian smearing was used with a value of 0.2 eV A (1 × 1 × 1) Γ-centred *k*-point grid was used to sample reciprocal space due to the large supercell size.

The electrolyte was classically modelled using the canonical ensemble MD. The one-dimensional hexagonal pores in the MOFs were filled with 1 M NEt_4BF_4 in acetonitrile assuming the density of the electrolyte is consistent with the bulk electrolyte. The free volume of the electrode is estimated using the Connolly surface area by employing a probe molecule with a kinetic diameter of 3.68 Å, equivalent to the kinetic diameter of N_2. Nosé–Hoover thermostat[14,15] was employed to set the temperature at 300 K, with a damping parameter of 100 fs. The OPLS-AA force field (FF)[16] was employed to describe the interatomic potential, based on the previous paper that the molecular solvation energy can be descried accurately from the DFT-CES[6] with OPLS-AA FF. For the Ni atom, the van der Waals (vdW) FF parameters for the Lennard-Jones (LJ) potential were carefully developed to accurately describe the interfacial interactions following the previous paper[17]. To determine the parameters, we obtained the binding energy curves between the electrolyte components and a fragment of $Ni_3(HITP)_2$ monolayer (SI Figure S10). The B3LYP-D3 functional with the LACVP**++ basis set, consisting of LANL2DZ effective core basis set for Ni, and standard Pople's 6-31G**++ basis set for other elements[18], using the NWChem software[19]. The external potential from the electrode in the QM region was set as follows: the DFT optimised structure and electrostatic potential obtained from the $(1 \times \sqrt{3})$ rect. model was repeated to fill the $(2 \times \sqrt{3})$ rect. model, resulting in an MD simulation cell dimension of 43.8 Å × 37.9 Å × 38.7 Å.

The chemical shift is calculated using the NWChem software[19] with the Gauge Including Atomic Orbitals (GIAO) formalism[20]. The calculated isotropic chemical shift, σ is scaled to computed the chemical shift, δ following the equation:

$$\delta = \frac{\text{intercept-}\sigma}{\text{-slope}} \qquad (1)$$

,where the *intercept* and *slope* values were used from the previous study[21,22]. The input geometries were selected using equilibrated QM/MM structures including BF_4^- and its first solvation shell. In case of adsorbed BF_4^-, $Ni_3(HITP)_2$ fragment is also included. The geometry optimisation is conducted with fixing the MOF fragment using DFT with B3LYP-D3 functional and the LACVP**++ basis set. Because of the computational cost, calculating the chemical shift with the explicit solvation model is only conducted for the BF_4^- in the bulk electrolyte and neutral $Ni_3(HITP)_2$. To study the chemical shift with charging effect, Conductor-like Screening Model (COSMO) implicit solvation method is employed[23] using acetonitrile as the solvent.

Electrochemical Characterisation

Electrode Preparation

$Ni_3(HITP)_2$ composite films were prepared using an existing published procedure, with acetylene black used as an additive to enhance electrode conductivity.[24] First, the electroactive components were sonicated together in ethanol (approx. 2 mL) for 15 minutes. The resultant slurry was added to a dispersion of 60 wt. % PTFE in water, in a few drops of ethanol. The mixture was stirred for 60 minutes, and over the subsequent period of 40 minutes incorporated into a film. Finally, the material was kneaded for 20 minutes, and rolled into a freestanding electrode film with a homemade aluminium rolling pin. The film was dried at 100 °C for at least 48 hours under dynamic vacuum in a vacuum oven. The resulting films had a final wt. % composition $Ni_3(HITP)_2$: acetylene black: PTFE of 85:10:5, and an approximate thickness of 250 µm.

Supercapacitor Assembly

Electrochemical measurements employed $Ni_3(HITP)_2$ composite electrodes cut from freestanding films in a N_2-filled glovebox using a 1/4" stainless-steel manual punching cutter (Hilka Tools). Whatman glass microfiber filter (GF/A) was used a separator (dried for 24 h, 100 °C), with two separators added to all cells.

For general electrochemical testing, symmetric supercapacitors were prepared as coin cells with two SS316 separator disks and one SS316 spring in CR2032 SS3016 coin cell cases (Cambridge Energy Solutions). A constant volume of electrolyte of 200 µL was added. A Compact Hydraulic Coin Cell Crimper (Cambridge Energy Solutions) was used to seal the coin cells in the glovebox at 80 kg cm^{-2} for 30 seconds.

For *ex-situ* NMR experiments, symmetric supercapacitors were first prepared in Swagelok PFA-820-3 union tube fittings with homemade stainless-steel plugs as current collectors. The two current collectors were sealed and 750 μL of electrolyte added before the Swagelok completely sealed. The cells were subsequently tightened with a spanner outside the glovebox to optimise their electrochemical performance.

Electrochemical Cell Characterisation

Biologic VSP-3e and SP-150 potentiostats were used to conduct electrochemical measurements. Gravimetric capacitance was determined from two-electrode coin cells assembled as above and calculated as recommended by Gittins *et al.*.[25] The 2nd discharge slope in a galvanostatic charge-discharge (GCD) experiment at a current density of 0.05 A g^{-1} and with a potential window of 1.0 V was employed in the calculation, using the mass of active electrode material (MOF).

Charging Experiments

Ex-Situ NMR Experiments

Ex-situ NMR spectroscopy experiments were performed on electrodes extracted from disassembled Swagelok supercapacitors. The cells were first cycled between cell voltages of 1 V and 0 V at 5 mV s^{-1} 30 times and left for a minimum of 12 hours for complete soaking of electrolyte. A constant voltage was held across the cell for 1 h with the current discharge recorded, and the cell returned immediately to the glovebox for disassembly. On supercapacitor disassembly, the two supercapacitor electrodes were packed simultaneously into two separate rotors to minimise acetonitrile electrolyte evaporation, unless otherwise specified.

Experiments were performed as detailed above, but with an MAS rate of 25 kHz required to achieved satisfactory resolution with the propylene carbonate electrolyte (**Figure S16d**).

Ex-Situ NMR Analysis

All spectra were fitted with Lorentzian peaks, and the resulting deconvoluted peaks integrated, using dmfit software. Parameters were not fixed between fits in order to obtain the best possible fits and avoid any bias as these are the first *ex-situ* NMR experiments performed on layered MOF supercapacitors.

For propylene carbonate electrolyte (**Figure 5a**), two peaks were sufficient to fit the in-pore and ex-pore environments, with negligible contribution to intensity from spinning sidebands (**SI Figure S4b**).

For acetonitrile electrolyte (**SI Figure S16b**), additional peak(s), intermediate in shift, were required for fitting features that are attributed to the fast exchange between in-pore and ex-pore environments. Isolated pockets of sharp free electrolyte peaks are in some cases additionally present and fitted in the spectra (**SI Figure S4c**). Moreover, any significant spinning sidebands were fitted using a CSA model (Haeberlen convention)

After integration of the in-pore peak (and their associated spinning sidebands), the integral values were converted to in-pore anion populations using the calibration curve in Figure S17.[26] For acetonitrile-based electrolyte, an upper bound for the in-pore anion population was determined by adding an additional contribution given by the fraction $\frac{\delta_{\text{exchange}}-\delta_{\text{free electrolyte}}}{\delta_{\text{in-pore}}-\delta_{\text{free electrolyte}}}$, that is, the proportion of the peak intermediate in chemical shift and considered to be in fast exchange between in-pore and ex-pore environments. The lower bound represents the in-pore anion population excluding any exchange contribution.

The in-pore chemical shift, and the calibrated in-pore anion population were plotted against the (specific) electronic charge on the electrode, determined from integration of the discharge current-time profile on EC-lab software (**Figures 5b-d**). The lines of best-fit plotted for both electrolytes, (**Figures 5b-d**) were determined by linear regression analysis. In Figures 5c and 5d, additional theoretical scenarios were plotted. These scenarios assume that electronic charge accumulation in the charged electrodes is perfectly counterbalanced by equal and opposite ionic charge accumulated during electrochemical double-layer formation at the electrode surfaces (i.e. in the in-pore environment). In (i), the “all cation” scenario, this denotes the expected change in the in-pore anion population with respect to electrode charge if only cation movement accounted for charge storage. This would require exclusively cation desorption on negative charging, and cation adsorption on positive charging. In this scenario, there is therefore no net change in the in-pore anion population with respect to electronic charge (a gradient of zero), corresponding to a 0% contribution of anion movement to charge storage. In contrast, in (ii) the “all anion” scenario, electronic charge is counterbalanced by ionic charge from anion adsorption on positive charging, and anion desorption on negative charging. This therefore represents the maximum possible change in in-pore anion population with respect to electronic charge and a 100% contribution of anion movement to charge storage.

The experimentally determined percentage anion contribution to charge storage was determined by $\frac{\text{gradient of experimental regression slope}}{\text{gradient of theoretical all anion slope}} \times 100$ %. The percentage error was determined by the percentage error in the experimental regression line, given by the 95% confidence interval. Under this definition: 100% corresponds to the “all anion” scenario; 0% corresponds to the “all cation” scenario; 50% corresponds to perfect ion exchange, *i.e.* equal amounts of counter-ion adsorption and co-ion desorption, and <0% corresponds to an “all cation” scenario in which some anions move in and out of the pores as ion pairs with the cations, thereby increasing the charge rather than counterbalancing it, and likewise >100% an “all anion” scenario in which cations also enter the pores alongside the anions (i.e. both anions and cations are adsorbed, albeit with an excess of anions).

Operando EQCM Experiments

Initially, a slurry was prepared by mixing 85 wt.% of the active material (MOF), 10 wt.% of carbon black, and 5 wt.% of polyvinylidene fluoride (PVDF) in N-Methyl-2-pyrrolidone (NMP) to achieve a concentration of 1 mg/mL. This slurry was then coated onto Au-coated quartz crystals with a basic oscillating frequency of 9 MHz (in air) using a spray gun, ensuring uniformity on the gold surface of the quartz sensor. 170 µL of the slurry was utilized for spray coating onto each quartz crystal. The distance between the spray gun and the quartz surface was maintained at approximately 20 cm during the coating process. The homogenously coated quartz crystal was subsequently vacuum-dried for 24 hours at 80°C before use. For the EQCM experiments, a three-electrode setup was employed, where the coated QCM quartz served as the working electrode, a platinum wire acted as the counter electrode, and Ag/AgCl was used as the reference electrode. These electrodes were positioned within a glassware container and immersed in the electrolyte solutions. All EQCM electrochemical measurements were conducted using a Maxtek RQCM system in conjunction with the Biologic potentiostat to enable simultaneous EQCM and electrochemical measurements.

In-Situ EQCM Analysis

The EQCM data was analyzed according to the Sauerbrey equation (Equation 2):

$$\Delta m = -C_f \Delta f \quad (2)$$

where Δm represents the change in mass of the surface coating, while C_f denotes the sensitivity factor of the quartz crystal sensor. The sensitivity factor for the bare quartz was determined through a copper deposition experiment carried out in an electrolyte solution containing 10 mM Cu_2SO_4 and 0.5 M H_2SO_4. Following stable cyclic voltammetry cycles, C_f was calculated as 6.98 ng·Hz^{-1} (or 5.43 ng·Hz^{-1} cm^{-2}, considering the surface area of the quartz crystal sensor is 1.28 cm^2). To ensure consistency in results, several cycles were performed before initiating EQCM measurements to establish stable and reproducible electrochemical signatures.

For calculating the molecular weight from Δm-ΔQ plots, the Faraday’s law was applied:

$$\frac{\Delta m}{\Delta Q} = \frac{M_W}{nF} \quad (3)$$

where Δm represents the change in mass of the surface coating, and ΔQ was the charges accumulated at the electrochemical interface, which was obtained by integrating current with time, n is the ion valence number and F is the Faraday constant (96 485 C/mol), then M_W is the experimentally obtained equivalent molecular weight. In our case, $\Delta m/\Delta Q$ is the slope of Δm-ΔQ plot, and n equals 1 as the charge carriers has the valence of 1.

Figure S1

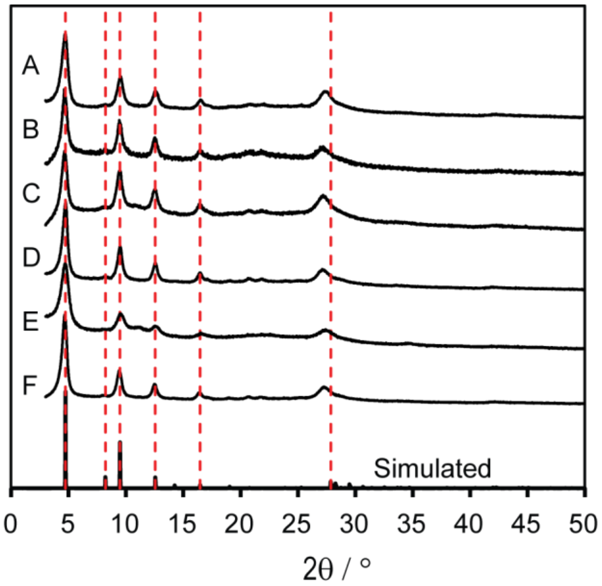

Figure S1: Experimental powder X-Ray diffraction (PXRD) of samples A-F used in this work, compared to simulated PXRD pattern of $Ni_3(HITP)_2$ a CIF file with eclipsed stacking of the 2D layers along the c axis. The comparison confirms identity and crystallinity of all samples in this work**.** Minimal variation was observed between samples. Spectra are scaled such that the maximum intensity is the same in each sample.

SI Figure S2

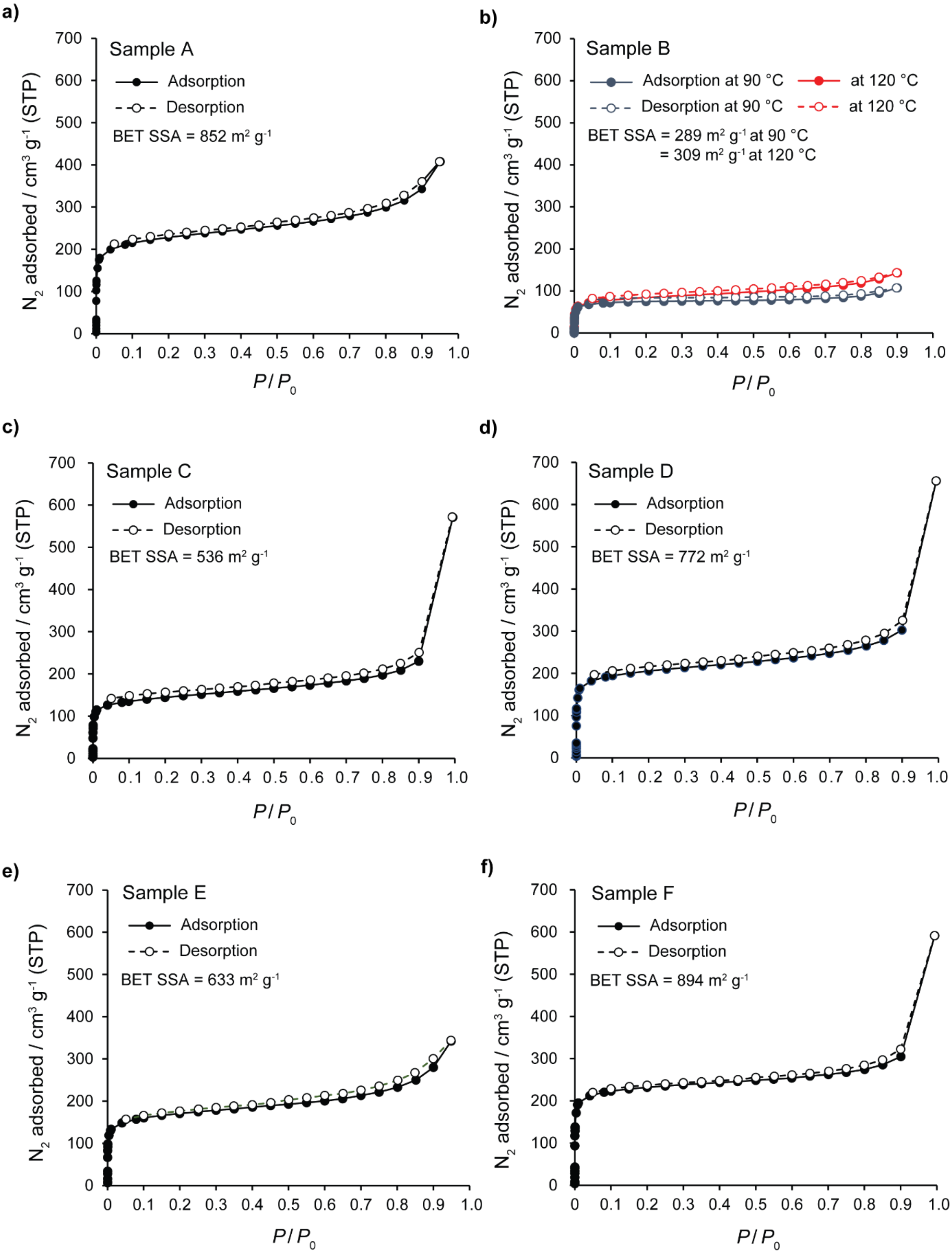

Figure S2: N_2 gas sorption isotherm @ 77 K of $Ni_3(HITP)_2$ samples A-F. Sample B was tested with outgassing temperatures of both 90 and 120 °C.

SI Table S2: MOF Sample Comparison

Sample	Degassing temp. / °C	BET SSA / $m^2 g^{-1}$	Fig. no.	Cg / $F g^{-1}$	In-pore ion population / $mmol g^{-1}$	Total Pore Volume of sample / $10^{-3} cm^3$	Maximum electrolyte volume added / $10^{-3} cm^3$
**A**	110	852	2,3,4	160	0.24	3.5	6.8
**B**	90, 120	289, 309	4	62	0.076	0.67	6.6
**C**	120	536	4	53	0.087	2.0	6.7
**D**	120	772	4	134	0.35	2.2	7.0
**E**	90	633	4	111	0.17	2.4	6.9
**F**	110	894	5,6	[redacted]	[redacted]	[redacted]	[redacted]

SI Figure S3

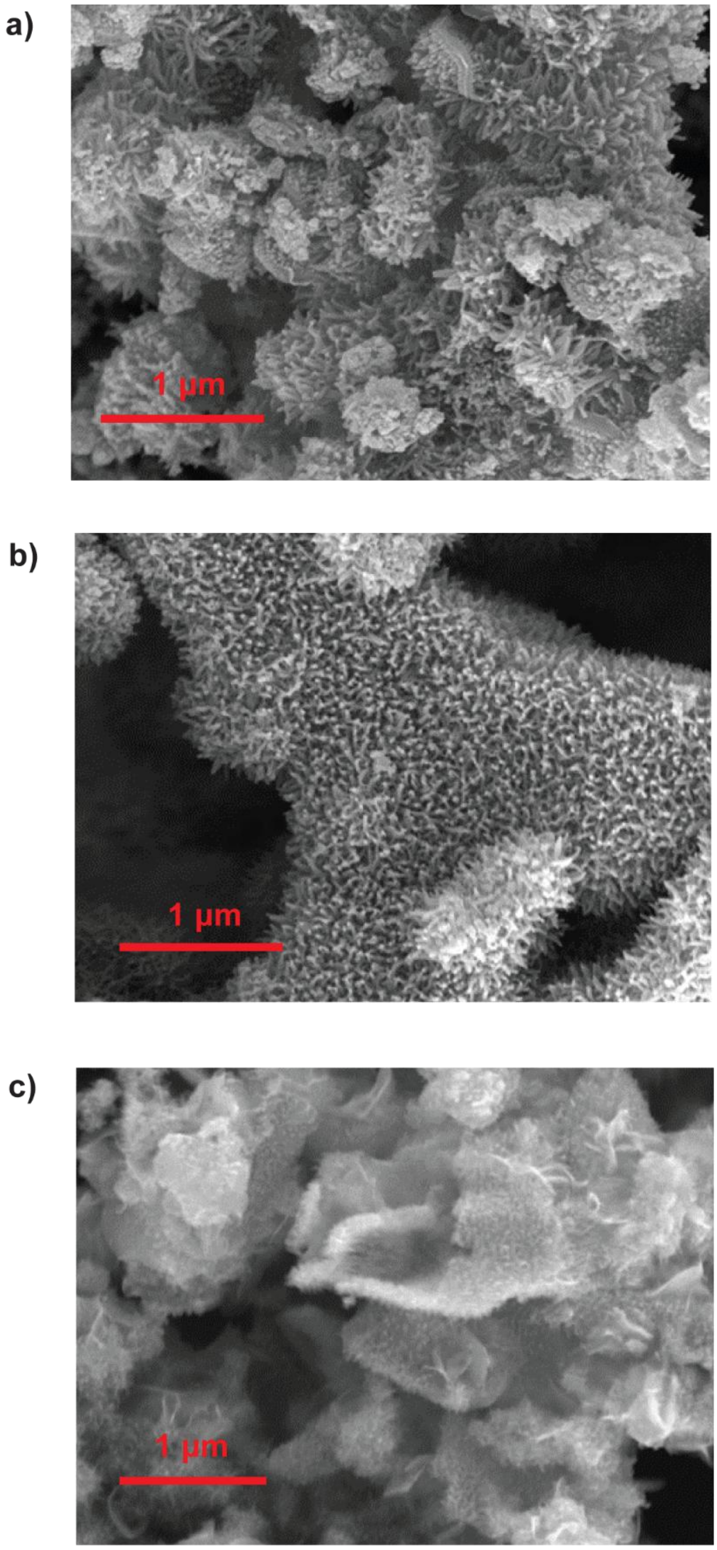

Figure S3: Scanning electron microscopy (SEM) image of $Ni_3(HITP)_2$ selected samples A, B and E studied in this work. In all cases the particles exhibit a rod-like morphology, with the rods agglomerating into larger microstructures. The field of view is between 3.8-4.0 µm for all samples.

SI Table S3: Predicted & Experimental Elemental Analysis Results

Element	Predicted / wt. %	Sample A / wt. %	Sample B / wt. %	Sample D / wt. %	Sample E / wt. %
Ni	22.0	15.1	13.1	12.2	15.3
C	54.0	44.4	50.3	41.7	46.5
H	3.0	3.9	3.6	3.6	3.3
N	21.0	15.1	18.3	15.7	16.5
Cl	0.0	3.2	3.2	3.1	2.8

SI Figure S4: Example NMR Deconvolution

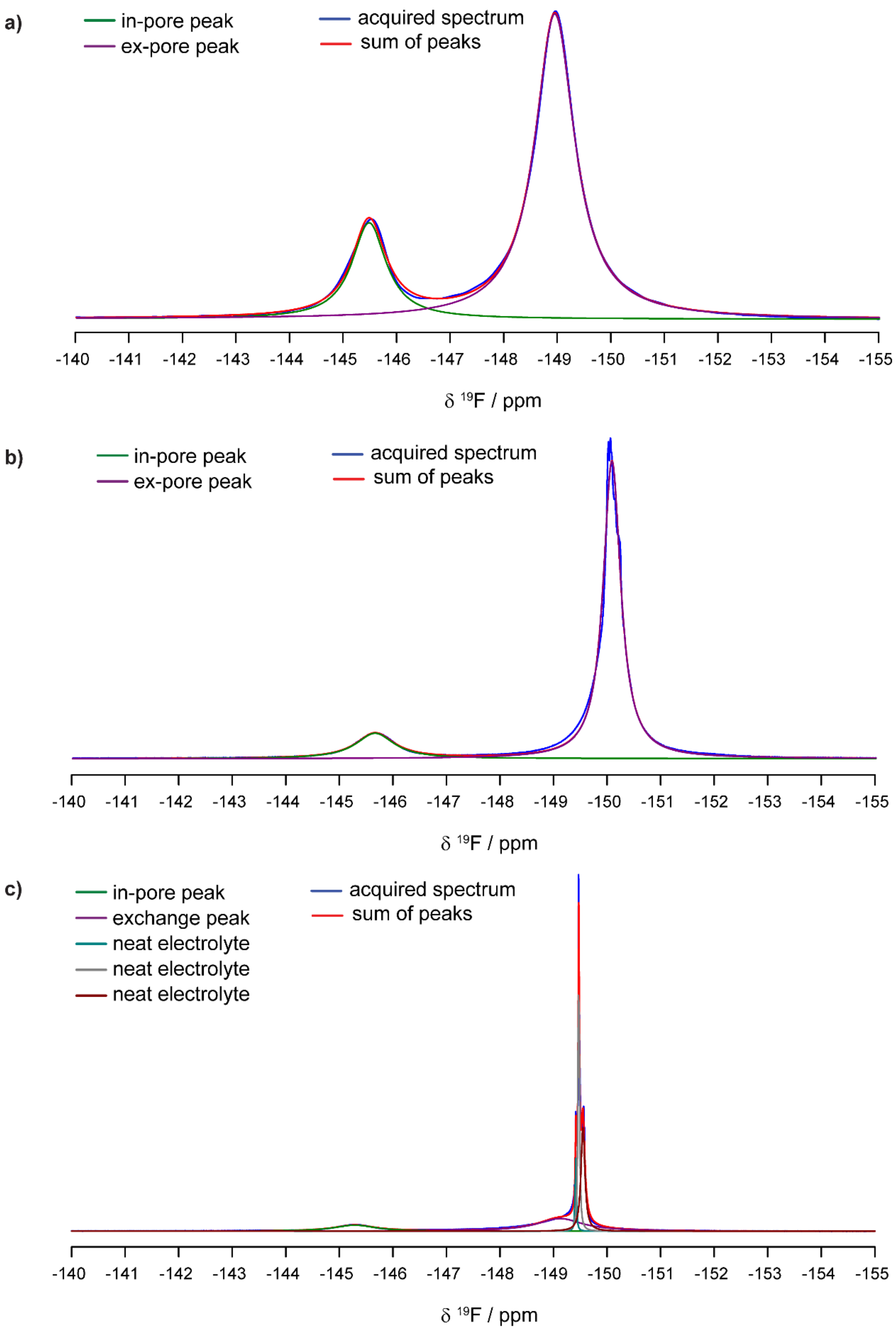

Figure S4: Example deconvolution of ^{19}F solid-state NMR of a) $Ni_3(HITP)_2$ sample A at a saturated loading of 1 M NEt_4BF_4/d_3ACN electrolyte (5 kHz MAS) from Figure 2a, b) *ex-situ* cell at 0 V of $Ni_3(HITP)_2$ sample F with 1 M NEt_4BF_4/PC (5 kHz MAS) from Figure 5a, and c) *ex-situ* cell at 0 V of $Ni_3(HITP)_2$ sample F with 1 M NEt_4BF_4/d_3ACN (25 kHz MAS), from SI Figure S16b.

SI Table S4 – ^{19}F NMR Anisotropy Fits for Electrolyte Soaked $Ni_3(HITP)_2$ Samples

Electrolyte	Sample	In-pore anisotropy / ppm	Ex-pore anisotropy / ppm
1 M NEt_4BF_4/d_3ACN	A (film)	18.6	10.6
	B (film)	18.6-21.2	13.3
	C (film)	23.9	17.3
	D (film)	13.3	6.6
	E (film)	21.2	10.6
	F (film)	14.6	9.3
1 M NEt_4TFSI/d_3ACN	A (powder)	15.9	8.0
1 M $NaSO_3CF_3$/DMF	A (powder)	14.6	4.0

SI Figure S5

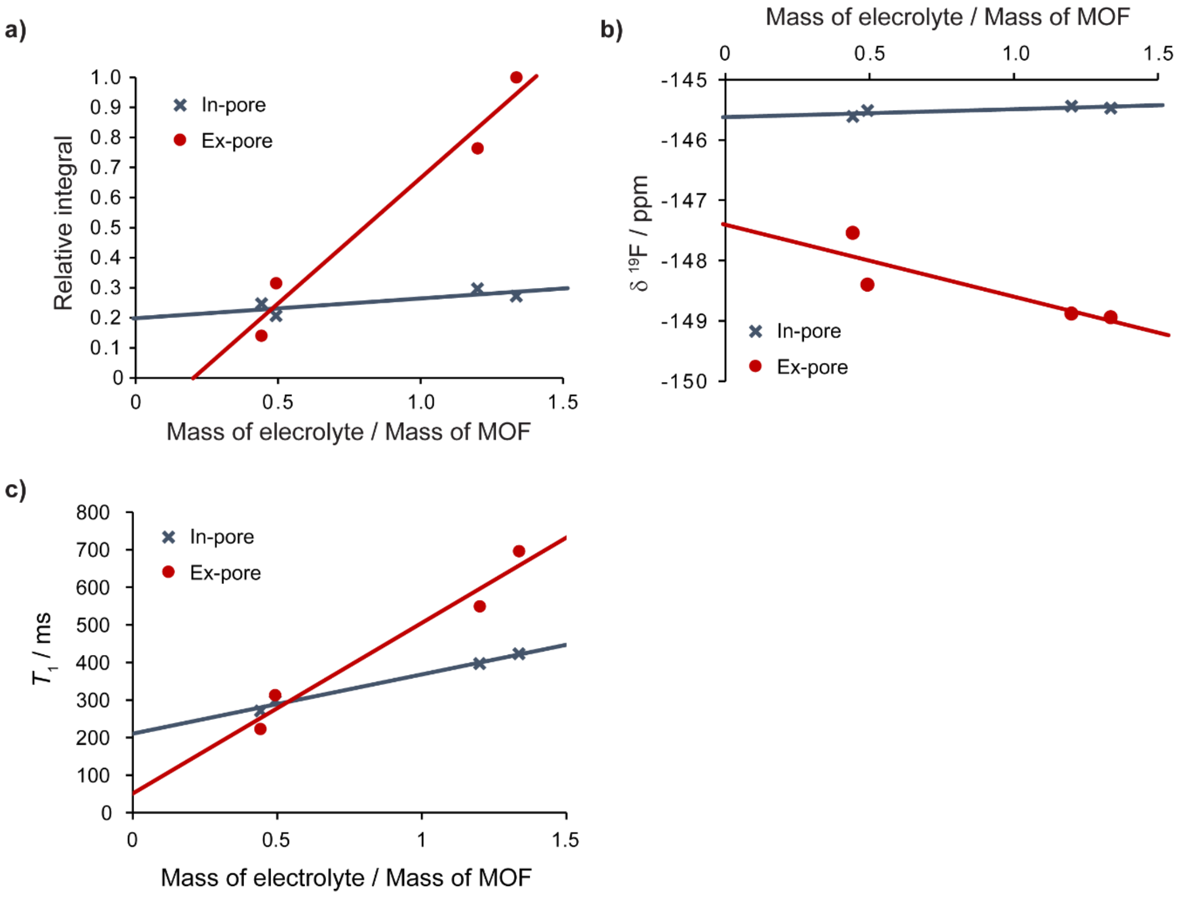

Figure S5: Results of fitting quantitative ^{19}F solid-state NMR spectra at 5 kHz MAS of powder $Ni_3(HITP)_2$ sample A at various loadings of 1 M NEt_4BF_4/d_3ACN electrolyte in Figure 2a. **a)** Relative integral of in-pore and ex-pore environments; , **b)** $\delta^{19}F$ of in-pore and ex-pore environments; and **c)** T_1 relaxation of in-pore and ex-pore environments as a function of $\frac{\text{Mass of electrolyte}}{\text{Mass of MOF}}$.

SI Figure S6

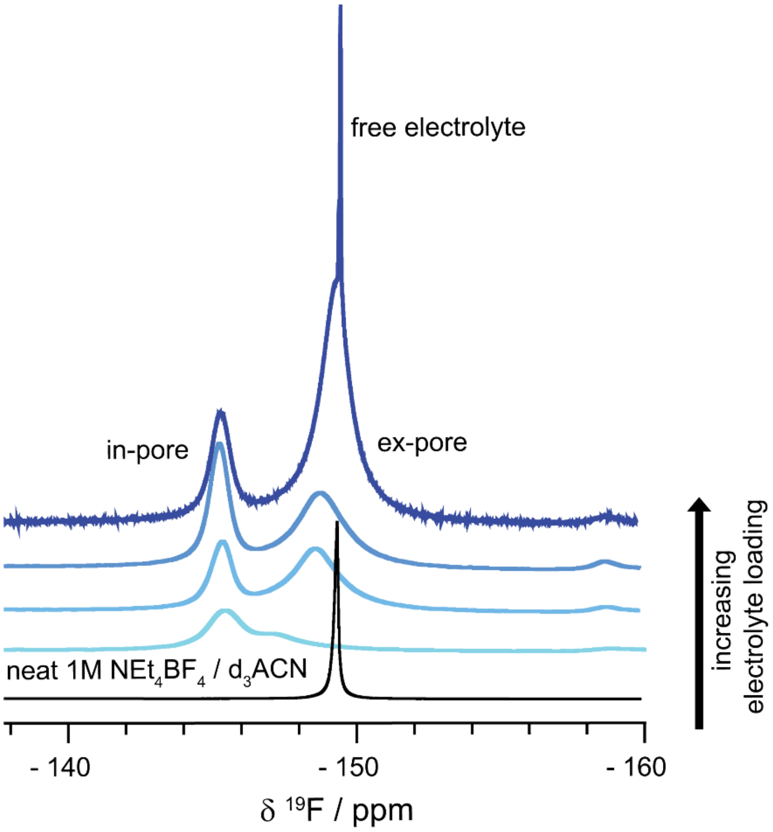

Figure S6: Quantitative ^{19}F solid-state NMR spectra at 5 kHz MAS of $Ni_3(HITP)_2$ sample A composite films at various loadings of 1 M NEt_4BF_4/d_3ACN electrolyte compared to ^{19}F NMR of the neat electrolyte. The darkening shade of blue indicates progressively higher electrolyte loadings, from light blue to dark blue: 0.3, 0.6, 1.0, 1.6 g of electrolyte per g of $Ni_3(HITP)_2$. Assignments are analogous to Figure 2a in the main text, with the additional appearance of a 'free electrolyte' environment superimposed on the ex-pore signal at the highest loading, as isolated pockets of electrolyte begin to form.

SI Figure S7

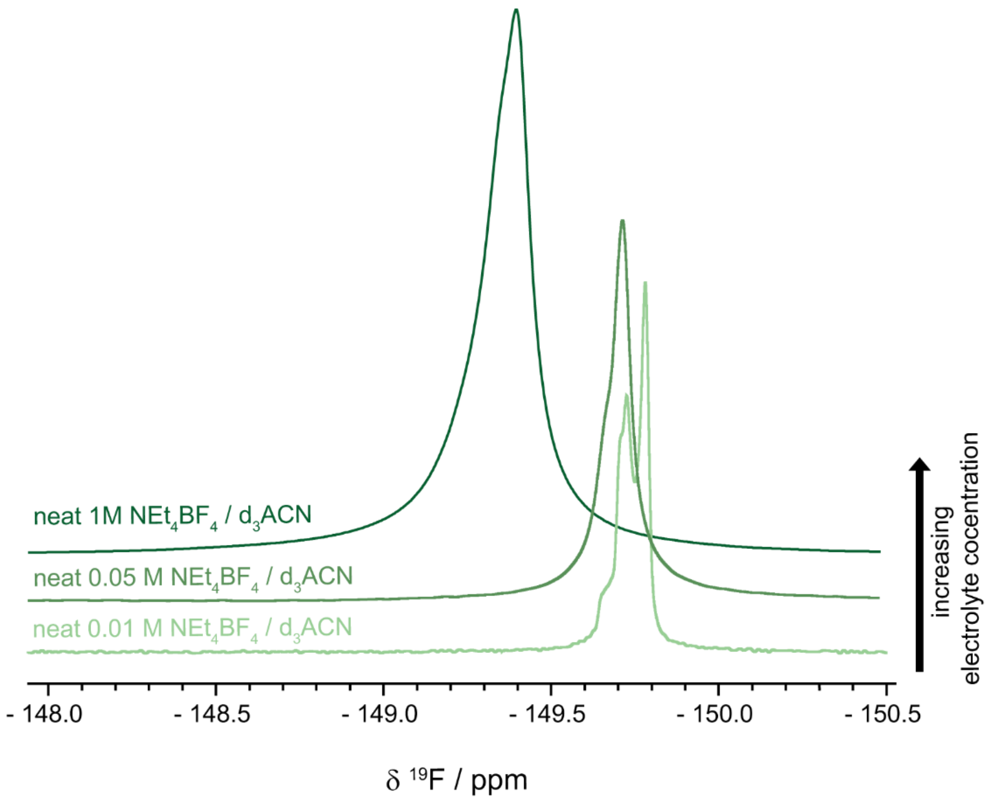

Figure S7: ^{19}F static NMR spectra of neat electrolytes at concentrations of 0.01, 0.05 and 1 M of NEt_4BF_4 in d_3ACN. The chemical shift is observed to change by only 0.39 ppm between these samples, making a concentration change unlikely to be responsible for the dominant contribution to the shift seen in the ex-pore peak for the electrolyte-soaked MOF samples.

SI Figure S8

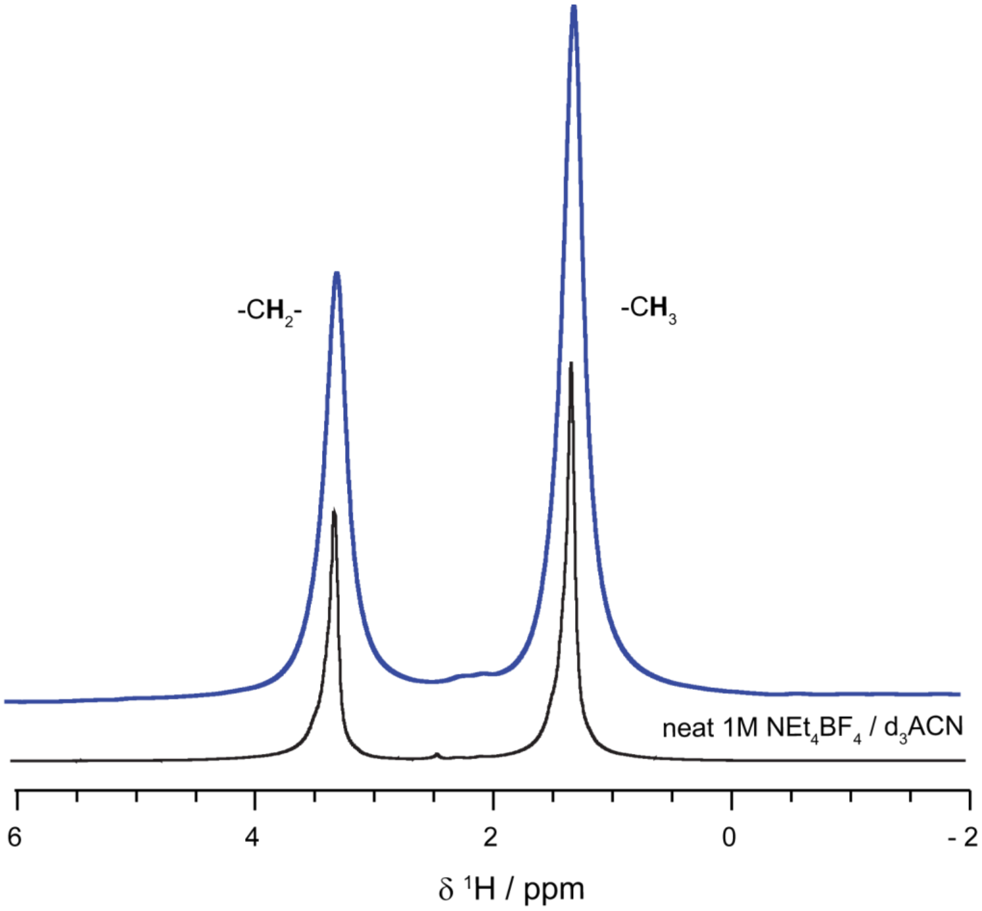

Figure S8: ^{1}H solid-state NMR spectra at 25 kHz MAS of powder $Ni_3(HITP)_2$ at a saturated loading of 1 M NEt_4BF_4/d_3ACN electrolyte compared to ^{1}H NMR of the neat electrolyte. The ^{1}H peaks are poorly resolved with minimal shift from the neat electrolyte, making fitting of the cation environments ambiguous.

SI Table S5: Experimental and Theoretical in-pore ^{19}F NMR Δδ

Electrolyte	NMR Nucleus		δ ^{19}F (in-pore) / ppm	δ ^{19}F (electrolyte) / ppm	Δδ ^{19}F / ppm
1 M NEt_4BF_4/d_3ACN	^{19}F	Experimental	−145.5	−149.4	+3.9
		Theoretical	−148.7	−152.1	+3.4
	^{11}B	Experimental	−0.5	−1.2	+0.7
		Theoretical	−0.8	−1.8	+1.0
1 M NEt_4TFSI/d_3ACN	^{19}F	Experimental	−76.0	−78.1	+2.1
1 M $NaSO_3CF_3/DMF$	^{19}F	Experimental	−75.5	−77.1	+1.6

SI Figure S9

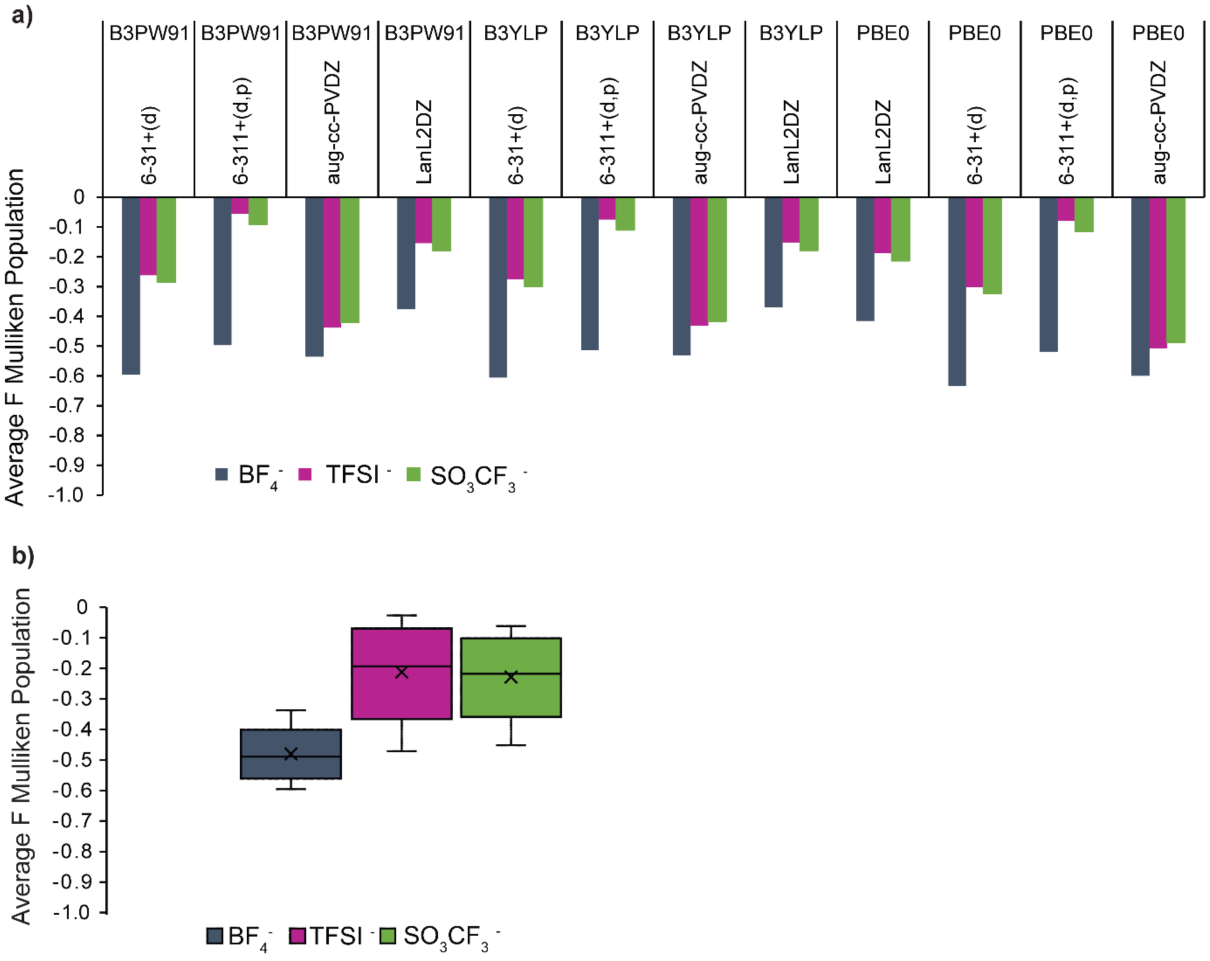

Figure S9: Gaussian calculations of average fluorine Mulliken population on BF_4^-, $TFSI^-$ and $SO_3CF_3^-$ singly charged anions **a)** With varying levels of theory and basis sets, in all cases the average Mulliken population on the fluorine atoms in the BF_4 anion is calculated to be more negative, implying a higher negative charge density, than other two cations **b)** Summary of results across the various basis sets and levels of theory.

SI Figure S10

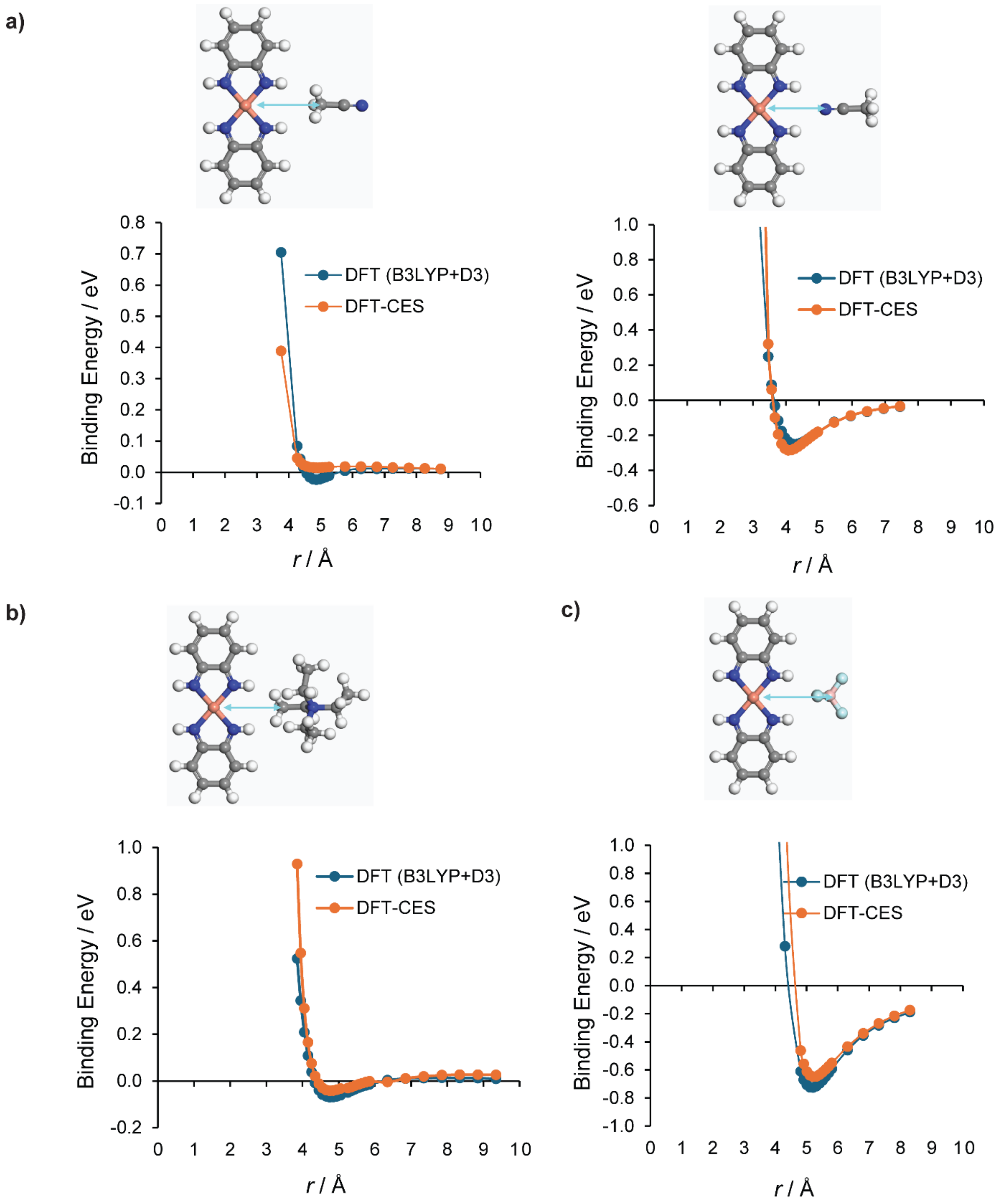

Figure S10: The binding energy from the DFT-CES method, which benchmark the DFT binding energy. $Ni_3(HITP)_2$ is represented as one Ni atom, four N atoms, four H atoms, and 2 benzene molecules. **a–c**, The binding energy between the MOF fragment and acetonitrile (**a**), BF_4^- (**b**), and NEt_4^+ (**c**) with multiple geometries. The Lennard-Jones potential parameter of which functional is $V(r)=\varepsilon\left[\left(\frac{R}{r}\right)^{12}-\left(\frac{R}{r}\right)^{6}\right]$ for the Ni atom is set as ε for 0.2 kcal mol^{-1} and R for 6.7 Å.

SI Figure S11

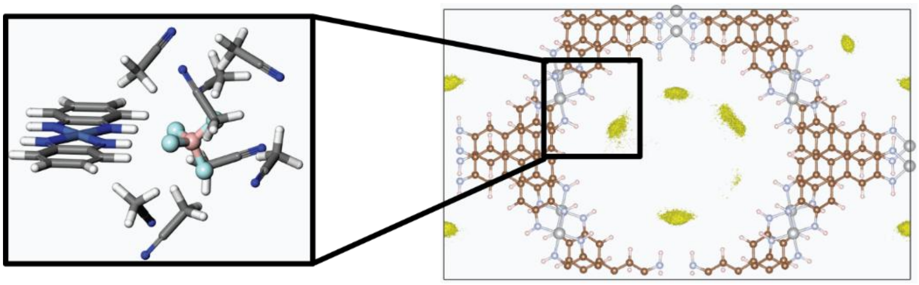

Figure S11: QM/MM simulated BF_4^- anion populations, highlighted in yellow, for 1 M NEt_4BF_4 electrolyte in $Ni_3(HITP)_2$, in the absence of an applied charge. The isosurface level is 0.003 e $bohr^{-3}$. Simulated anion population symmetry is not fully maintained in the 5 ns sampling time used here due to its strong binding affinity to the electrode.

SI Figure S12

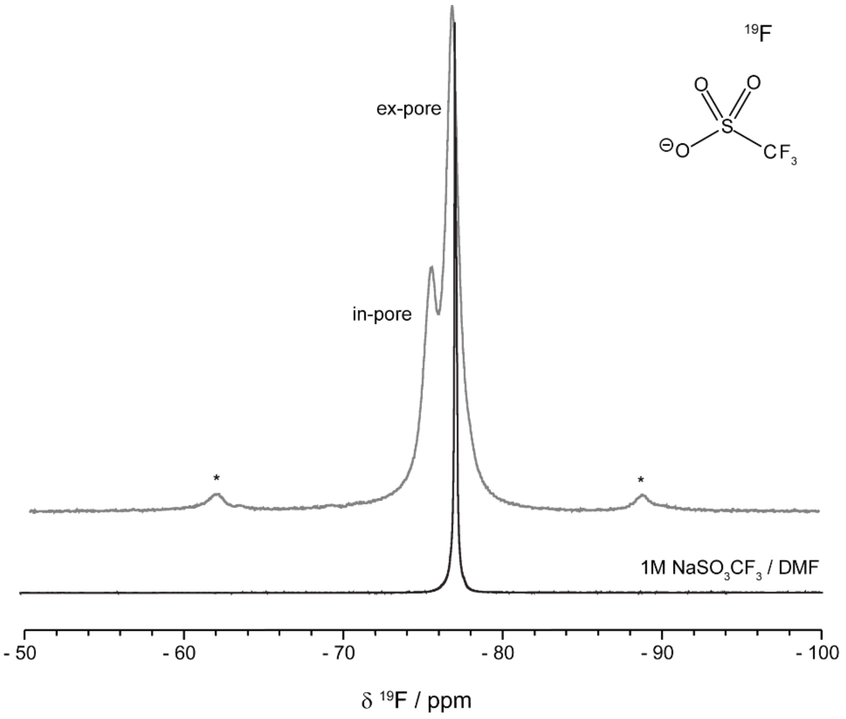

Figure S12: ^{19}F solid-state NMR spectra at 5 kHz MAS of powder $Ni_3(HITP)_2$ Sample A soaked with 1 M $NaSO_3CF_3$ / DMF compared to neat electrolyte. Same spectrum as in Figure 3e but zoomed out to see in-pore spinning sidebands marked with *.

SI Figure S13

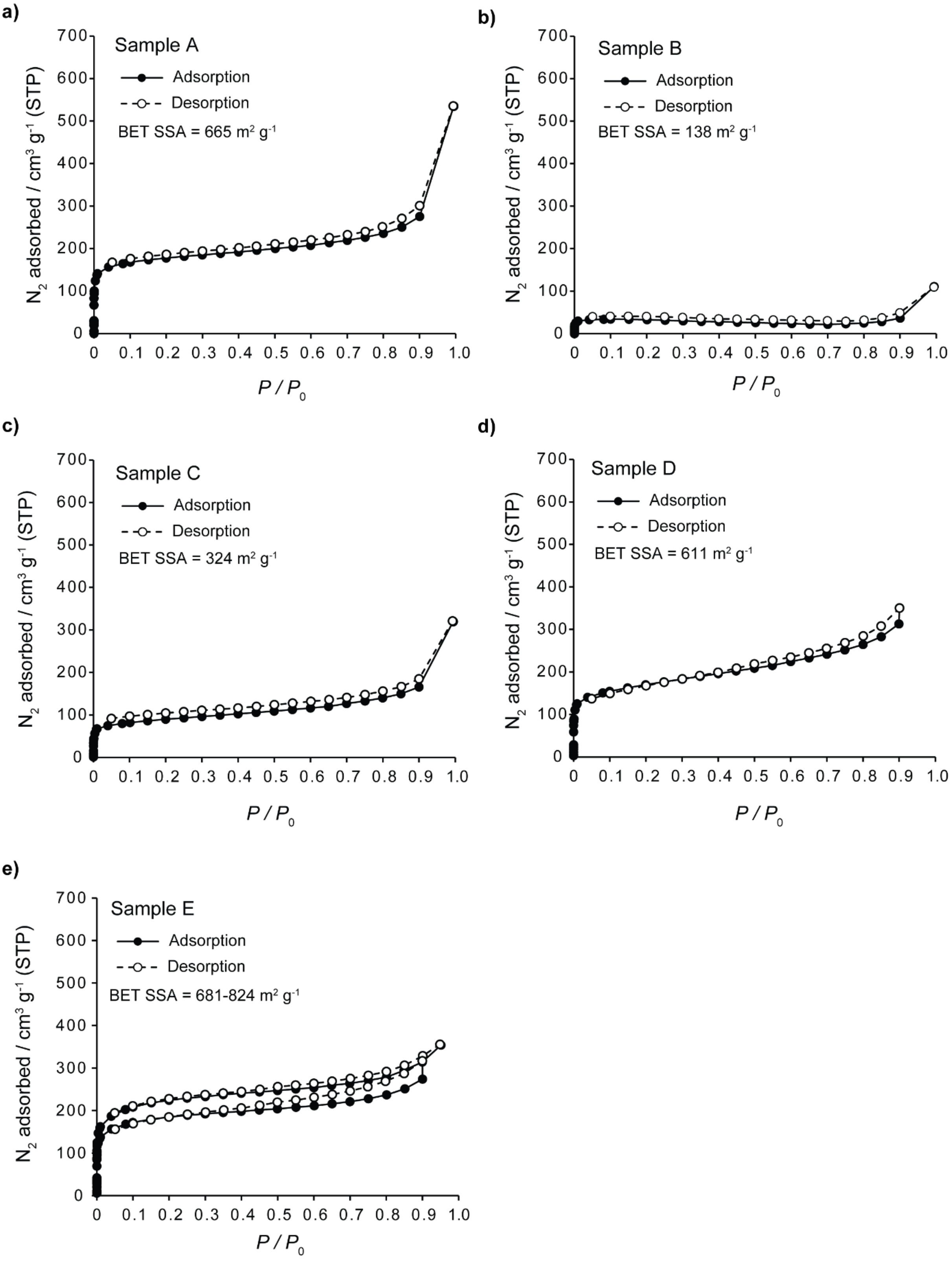

Figure S13: N_2 gas sorption isotherm @ 77 K of $Ni_3(HITP)_2$ 85 wt. % composite films samples A-E. Sample E gave rise to an anomalously high BET SSA, so a repeat measurement on another portion of the sample was taken.

SI Figure S14

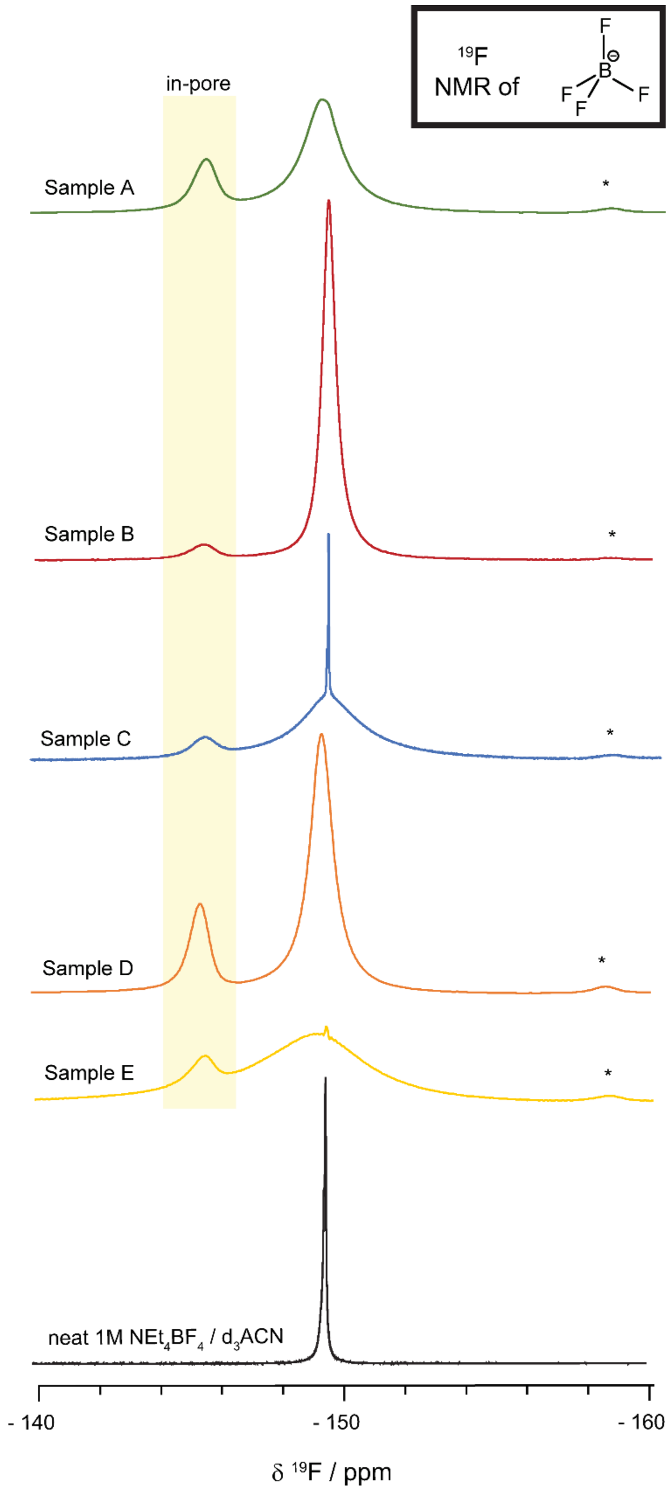

Figure S14: Quantitative ^{19}F solid-state NMR spectra at 5 kHz MAS of composite film of $Ni_3(HITP)_2$ Samples A-E soaked with 1 M NEt_4BF_4 / d_3ACN compared to neat electrolyte, at a high loading to saturate the MOF porosity (SI Table S2). In-pore peak is highlighted, showing variations in intensity but the peak shape of the ex-pore (and neat electrolyte) environment(s) also vary. Spinning sidebands at 5 kHz separation identified with *.

SI Figure S15

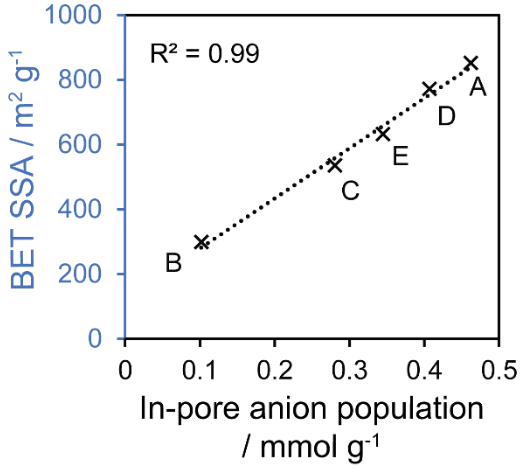

Figure S15: Correlation between BET SSA of powder MOF samples and in-pore anion population for the five $Ni_3(HITP)_2$ composite film samples studied in Figure 4.

SI Figure S16

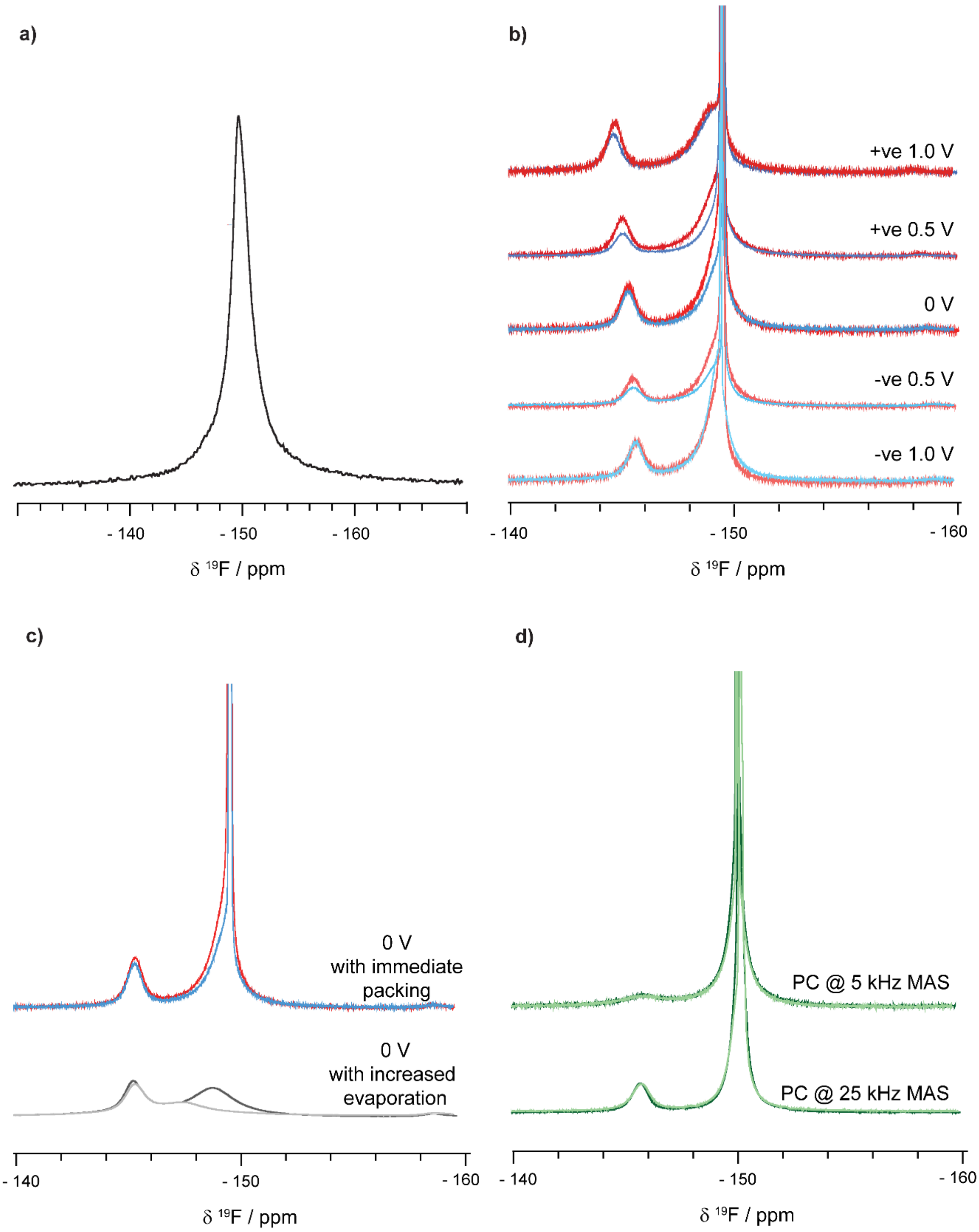

Figure S16: ^{19}F solid-state NMR spectra of charged (*ex-situ*) $Ni_3(HITP)_2$ **a)** static spectrum, in 1 M NEt_4BF_4/d_3ACN electrolyte; positive electrode held at 1.5 V for 1 hour. Spectrum shows poor resolution of environments under static conditions, hence *in-situ* experiments were not feasible. **b)** at 5 kHz MAS, in 1 M NEt_4BF_4/d_3ACN electrolyte; electrodes held at various voltages for 1 hour, with two repeats of each electrode shown. Variability between repeats is attributed to evaporation of the solvent from packing time. **c)** at 5 kHz MAS, in 1 M NEt_4BF_4/d_3ACN electrolyte; electrodes held at 0 V for 1 hour, with two repeats of each electrode shown. Immediate packing is compared to electrodes where electrodes were deliberately left for the solvent to evaporate, demonstrating the resulting dramatic reduction in intensity and change in spectral appearance. **d)** at 5 kHz and 25 kHz MAS, in 1 M NEt_4BF_4/PC electrolyte; electrodes held at 0 V for 1 hour, with two repeats of the electrode shown. Improved resolution of the spectra at 25 kHz emphasise why this MAS rate was required for the propylene carbonate solvent.

SI Figure S17

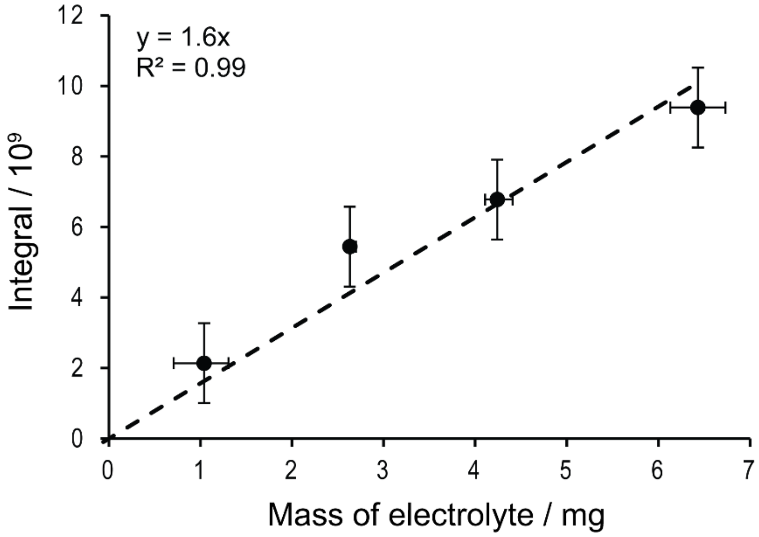

Figure S17: Calibration curve of total integral from NMR experiments against mass of electrolyte added for soaked film samples of $Ni_3(HITP)_2$ with known electrolyte loading. The calibration from the line of regression was then used to calculate the in-pore ion population for charged (*ex-situ*) $Ni_3(HITP)_2$ samples from the integral of the in-pore peak in NMR experiments.

SI Table S6: Simulations of δ ^{19}F for BF_4^- anion adsorbed on Ni MOF fragment

MOF fragment charge / *e*	ESP charge / *e*	*r* / Å	δ ^{19}F / ppm	$\Delta\delta$ ^{19}F / ppm
2.00	−0.98	4.81	−170.81	+9.58
0.00	−1.02	4.98	−172.71	+7.68
−2.00	−1.05	5.22	−175.09	+5.30

SI Figure S18

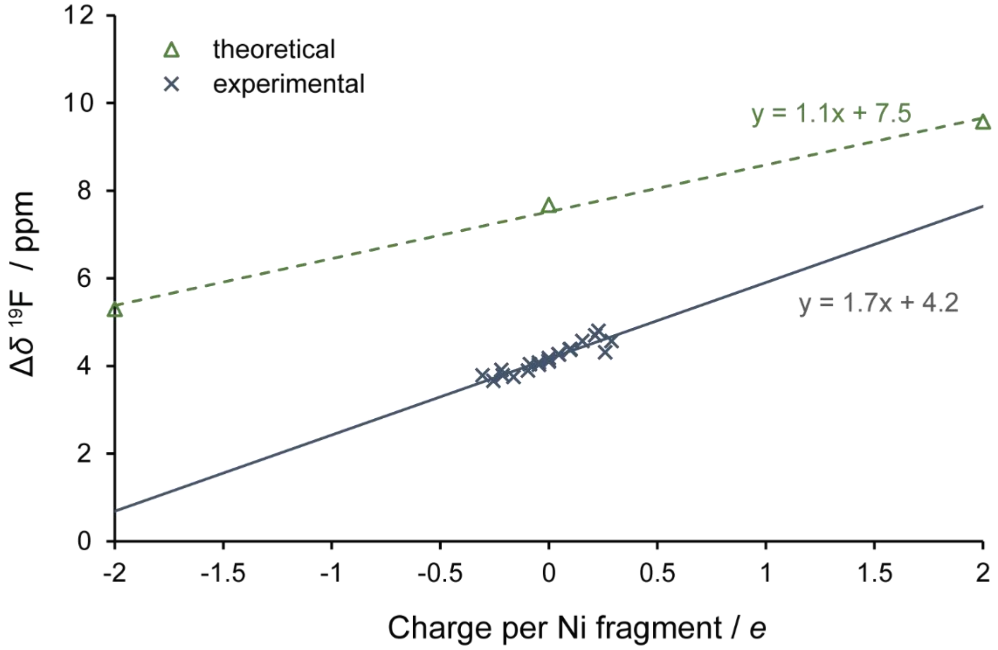

Figure S18: Comparison of experimental $\Delta\delta$ ^{19}F with simulated values for a BF_4^- anion adsorbed to a Ni MOF fragment with implicit solvation. Whilst the theoretical values overestimate $\Delta\delta$ at the charges used experimentally, and underestimate the slope of the relationship, the positive correlation between $\Delta\delta$ and the charge is in qualitative agreement.

SI Figure S19

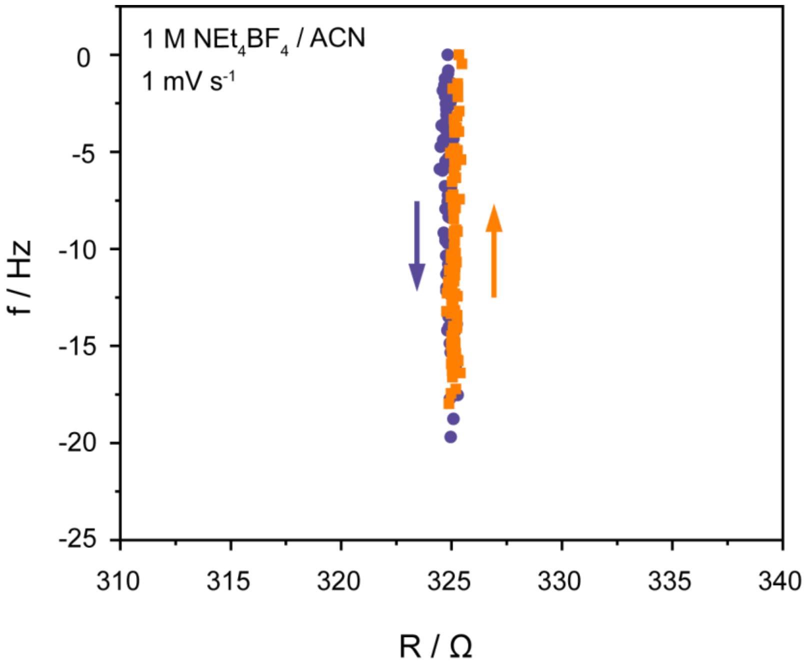

Figure S19: Measured frequency against resistance during electrochemical cycling with a potential window of +200 mV to +500 mV at 1mV s^{-1}. Negligible change in motional resistance was observed, thus validating the gravimetric analysis of the frequency change using the Sauerbrey equation.

SI Figure S20

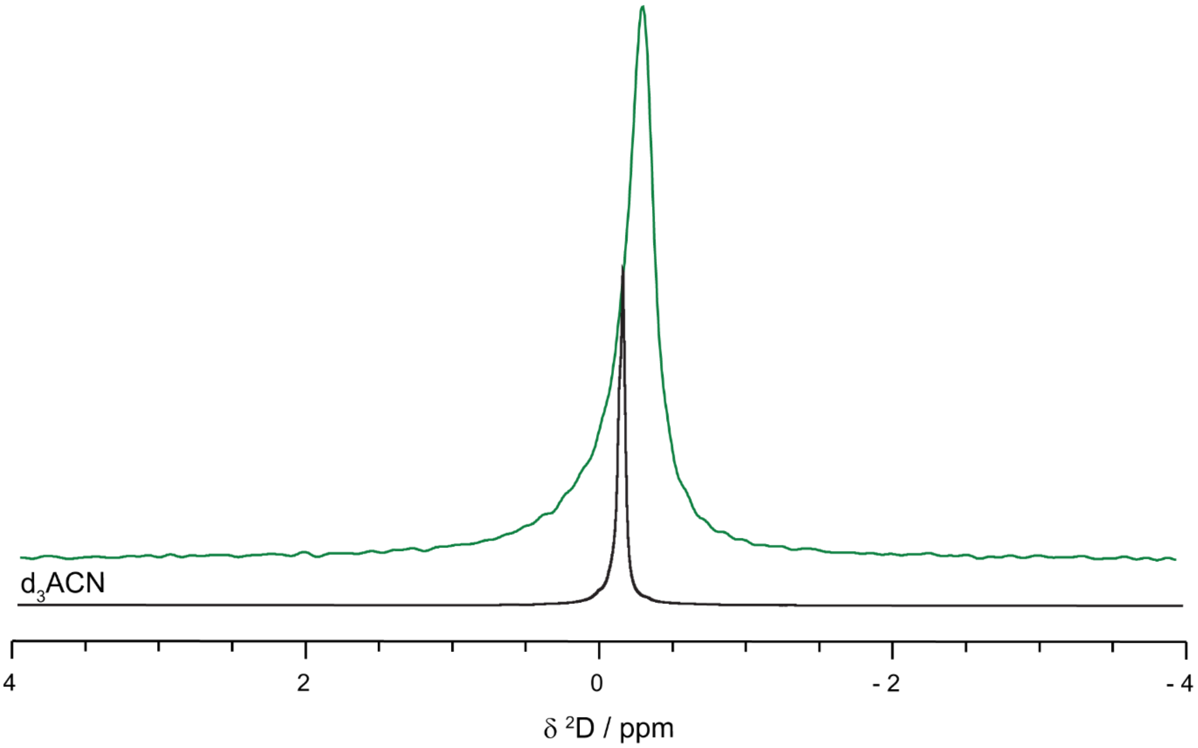

Figure S20: ^{2}D solid-state NMR spectrum at 25 kHz MAS rate of $Ni_3(HITP)_2$ Sample B soaked in d_3ACN compared to neat solvent. The broad feature is not well resolved into different chemical environments.

References

(1) Sheberla, D.; Sun, L.; Blood-Forsythe, M. A.; Er, S.; Wade, C. R.; Brozek, C. K.; Aspuru-Guzik, A.; Dincă, M. High Electrical Conductivity in Ni_3 (2,3,6,7,10,11-Hexaiminotriphenylene) $_2$, a Semiconducting Metal–Organic Graphene Analogue. *J. Am. Chem. Soc.* **2014**, *136* (25), 8859–8862. https://doi.org/10.1021/ja502765n.

(2) Thommes, M.; Kaneko, K.; Neimark, A. V.; Olivier, J. P.; Rodriguez-Reinoso, F.; Rouquerol, J.; Sing, K. S. W. Physisorption of Gases, with Special Reference to the Evaluation of Surface Area and Pore Size Distribution (IUPAC Technical Report). *Pure Appl. Chem.* **2015**, *87* (9–10), 1051–1069. https://doi.org/10.1515/pac-2014-1117.

(3) Kim, T.; Ideta, K.; Jung, D.; Saito, K.; Park, J.-I.; Rhee, C. K.; Miyawaki, J.; Mochida, I.; Yoon, S.-H. Quantitative Analysis of BF4− Ions Infiltrated into Micropores of Activated Carbon Fibers Using Nuclear Magnetic Resonance. *RSC Adv.* **2014**, *4* (32), 16726. https://doi.org/10.1039/c3ra46436a.

(4) Griffin, J. M.; Forse, A. C.; Grey, C. P. Solid-State NMR Studies of Supercapacitors. *Solid State Nucl. Magn. Reson.* **2016**, *74–75*, 16–35. https://doi.org/10.1016/j.ssnmr.2016.03.003.

(5) Frisch, M. J.; Trucks, G. W.; Schlegel, H. B.; Scuseria, G. E.; Robb, M. A.; Cheeseman, J. R.; Scalmani, G.; Barone, V.; Petersson, G. A.; Nakatsuji, H.; Li, X.; Caricato, M.; Marenich, A. V.; Bloino, J.; Janesko, B. G.; Gomperts, R.; Mennucci, B.; Hratchian, H. P.; Ortiz, J. V.; Izmaylov, A. F.; Sonnenberg, J. L.; Williams-Young, D.; Ding, F.; Lipparini, F.; Egidi, F.; Goings, J.; Peng, B.; Petrone, A.; Henderson, T.; Ranasinghe, D.; Zakrzewski, V. G.; Gao, J.; Rega, N.; Zheng, G.; Liang, W.; Hada, M.; Ehara, M.; Toyota, K.; Fukuda, R.; Hasegawa, J.; Ishida, M.; Nakajima, T.; Honda, Y.; Kitao, O.; Nakai, H.; Vreven, T.; Throssell, K.; Montgomery, J. A., Jr.; Peralta, J. E.; Ogliaro, F.; Bearpark, M. J.; Heyd, J. J.; Brothers, E. N.; Kudin, K. N.; Staroverov, V. N.; Keith, T. A.; Kobayashi, R.; Normand, J.; Raghavachari, K.; Rendell, A. P.; Burant, J. C.; Iyengar, S. S.; Tomasi, J.; Cossi, M.; Millam, J. M.; Klene, M.; Adamo, C.; Cammi, R.; Ochterski, J. W.; Martin, R. L.; Morokuma, K.; Farkas, O.; Foresman, J. B.; Fox, D. J. Gaussian, Inc., Wallingford CT, 2016. Gaussian 16, Revision C.01.

(6) Lim, H.-K.; Lee, H.; Kim, H. A Seamless Grid-Based Interface for Mean-Field QM/MM Coupled with Efficient Solvation Free Energy Calculations. *J. Chem. Theory Comput.* **2016**, *12* (10), 5088–5099. https://doi.org/10.1021/acs.jctc.6b00469.

(7) Giannozzi, P.; Baroni, S.; Bonini, N.; Calandra, M.; Car, R.; Cavazzoni, C.; Ceresoli, D.; Chiarotti, G. L.; Cococcioni, M.; Dabo, I.; Dal Corso, A.; de Gironcoli, S.; Fabris, S.; Fratesi, G.; Gebauer, R.; Gerstmann, U.; Gougoussis, C.; Kokalj, A.; Lazzeri, M.; Martin-Samos, L.; Marzari, N.; Mauri, F.; Mazzarello, R.; Paolini, S.; Pasquarello, A.; Paulatto, L.; Sbraccia, C.; Scandolo, S.; Sclauzero, G.; Seitsonen, A. P.; Smogunov, A.; Umari, P.; Wentzcovitch, R. M. QUANTUM ESPRESSO: A Modular and Open-Source Software Project for Quantum Simulations of Materials. *J. Phys. Condens. Matter* **2009**, *21* (39), 395502. https://doi.org/10.1088/0953-8984/21/39/395502.

(8) Plimpton, S. Fast Parallel Algorithms for Short-Range Molecular Dynamics. *J. Comput. Phys.* **1995**, *117*, 1–19. https://doi.org/10.1006/jcph.1995.1039.

(9) Sheberla, D.; Sun, L.; Blood-Forsythe, M. A.; Er, S.; Wade, C. R.; Brozek, C. K.; Aspuru-Guzik, A.; Dincă, M. High Electrical Conductivity in Ni_3 (2,3,6,7,10,11-Hexaiminotriphenylene)$_2$, a Semiconducting Metal–Organic Graphene Analogue. *J. Am. Chem. Soc.* **2014**, *136* (25), 8859–8862. https://doi.org/10.1021/ja502765n.

(10) Perdew, J. P.; Burke, K.; Ernzerhof, M. Generalized Gradient Approximation Made Simple. *Phys. Rev. Lett.* **1996**, *77* (18), 3865–3868. https://doi.org/10.1103/PhysRevLett.77.3865.

(11) Grimme, S.; Antony, J.; Ehrlich, S.; Krieg, H. A Consistent and Accurate *Ab Initio* Parametrization of Density Functional Dispersion Correction (DFT-D) for the 94 Elements H-Pu. *J. Chem. Phys.* **2010**, *132* (15), 154104. https://doi.org/10.1063/1.3382344.

(12) Rosen, A. S.; Notestein, J. M.; Snurr, R. Q. Comparing GGA, GGA+U, and Meta-GGA Functionals for Redox-Dependent Binding at Open Metal Sites in Metal–Organic Frameworks. *J. Chem. Phys.* **2020**, *152* (22), 224101. https://doi.org/10.1063/5.0010166.

(13) Blöchl, P. E. Projector Augmented-Wave Method. *Phys. Rev. B* **1994**, *50* (24), 17953–17979. https://doi.org/10.1103/PhysRevB.50.17953.

(14) Nosé, S. A Unified Formulation of the Constant Temperature Molecular Dynamics Methods. *J. Chem. Phys.* **1984**, *81* (1), 511–519. https://doi.org/10.1063/1.447334.

(15) Hoover, W. G. Canonical Dynamics: Equilibrium Phase-Space Distributions. *Phys. Rev. A* **1985**, *31* (3), 1695–1697. https://doi.org/10.1103/PhysRevA.31.1695.

(16) Jorgensen, W. L.; Maxwell, D. S.; Tirado-Rives, J. Development and Testing of the OPLS All-Atom Force Field on Conformational Energetics and Properties of Organic Liquids. *J. Am. Chem. Soc.* **1996**, *118* (45), 11225–11236. https://doi.org/10.1021/ja9621760.

(17) Shin, S.-J.; Gittins, J. W.; Golomb, M. J.; Forse, A. C.; Walsh, A. Microscopic Origin of Electrochemical Capacitance in Metal–Organic Frameworks. *J. Am. Chem. Soc.* **2023**, *145* (26), 14529–14538. https://doi.org/10.1021/jacs.3c04625.

(18) Hay, P. J.; Wadt, W. R. *Ab Initio* Effective Core Potentials for Molecular Calculations. Potentials for K to Au Including the Outermost Core Orbitals. *J. Chem. Phys.* **1985**, *82* (1), 299–310. https://doi.org/10.1063/1.448975.

(19) Valiev, M.; Bylaska, E. J.; Govind, N.; Kowalski, K.; Straatsma, T. P.; Van Dam, H. J. J.; Wang, D.; Nieplocha, J.; Apra, E.; Windus, T. L.; de Jong, W. A. NWChem: A Comprehensive and Scalable Open-Source Solution

for Large Scale Molecular Simulations. *Comput. Phys. Commun.* **2010**, *181* (9), 1477–1489. https://doi.org/10.1016/j.cpc.2010.04.018.
(20) Dupuis, M. New Integral Transforms for Molecular Properties and Application to a Massively Parallel GIAO-SCF Implementation. *Comput. Phys. Commun.* **2001**, *134* (2), 150–166. https://doi.org/10.1016/S0010-4655(00)00195-8.
(21) Benassi, E. An Inexpensive Density Functional Theory-Based Protocol to Predict Accurate 19F-NMR Chemical Shifts. *J. Comput. Chem.* **2022**, *43* (3), 170–183. https://doi.org/10.1002/jcc.26780.
(22) Gao, P.; Wang, X.; Huang, Z.; Yu, H. 11B NMR Chemical Shift Predictions via Density Functional Theory and Gauge-Including Atomic Orbital Approach: Applications to Structural Elucidations of Boron-Containing Molecules. *ACS Omega* **2019**, *4* (7), 12385–12392. https://doi.org/10.1021/acsomega.9b01566.
(23) Klamt, A.; Schüürmann, G. COSMO: A New Approach to Dielectric Screening in Solvents with Explicit Expressions for the Screening Energy and Its Gradient. *J Chem Soc Perkin Trans 2* **1993**, No. 5, 799–805. https://doi.org/10.1039/P29930000799.
(24) Gittins, J. W.; Balhatchet, C. J.; Chen, Y.; Liu, C.; Madden, D. G.; Britto, S.; Golomb, M. J.; Walsh, A.; Fairen-Jimenez, D.; Dutton, S. E.; Forse, A. C. Insights into the Electric Double-Layer Capacitance of Two-Dimensional Electrically Conductive Metal–Organic Frameworks. *J. Mater. Chem. A* **2021**, *9* (29), 16006–16015. https://doi.org/10.1039/D1TA04026J.
(25) Gittins, J. W.; Chen, Y.; Arnold, S.; Augustyn, V.; Balducci, A.; Brousse, T.; Frackowiak, E.; Gómez-Romero, P.; Kanwade, A.; Köps, L.; Jha, P. K.; Lyu, D.; Meo, M.; Pandey, D.; Pang, L.; Presser, V.; Rapisarda, M.; Rueda-García, D.; Saeed, S.; Shirage, P. M.; Ślesiński, A.; Soavi, F.; Thomas, J.; Titirici, M.-M.; Wang, H.; Xu, Z.; Yu, A.; Zhang, M.; Forse, A. C. Interlaboratory Study Assessing the Analysis of Supercapacitor Electrochemistry Data. *J. Power Sources* **2023**, *585*, 233637. https://doi.org/10.1016/j.jpowsour.2023.233637.
(26) Bak, M.; Rasmussen, J. T.; Nielsen, N. C. SIMPSON: A General Simulation Program for Solid-State NMR Spectroscopy. *J. Magn. Reson.* **2000**, *147* (2), 296–330. https://doi.org/10.1006/jmre.2000.2179.
